# TaPYL-mediated chemical activation of ABA signaling in wheat enhances water productivity and reinforces carbohydrate reserves

**DOI:** 10.1007/s00425-026-05216-3

**Published:** 2026-11-10

**Authors:** Mar Bono, Miriam Marín-Sanz, Estefanía Uberegui, Javier Calvo, Cristian Mayordomo, Armando Albert, Javier Forment, Francisco Barro, Rosa Morcuende, Juan B. Arellano, Rubén Vicente, Pedro L. Rodriguez

**Affiliations:** 1 Instituto de Biología Molecular y Celular de Plantas (IBMCP), Consejo Superior de Investigaciones Científicas-Universidad Politécnica de Valencia, ES-46022 Valencia, Spain; 2 Instituto Maimónides de Investigación Biomédica de Córdoba (IMIBIC)- Hospital Universitario Reina Sofía, Córdoba, Spain; 3 Instituto de Recursos Naturales y Agrobiología de Salamanca (IRNASA), Consejo Superior de Investigaciones Científicas (CSIC), Salamanca, Spain; 4 Instituto de Química-Física “Blas Cabrera” (IQF) Consejo Superior de Investigaciones Científicas, ES-28006 Madrid, Spain; 5 Instituto de Agricultura Sostenible (IAS), Consejo Superior de Investigaciones Científicas (CISC), Córdoba, Spain

**Keywords:** ABA receptor, ISB09, Water use efficiency, Fructan, Starch, Raffinose

## Abstract

**Main conclusion:**

Chemical activation of ABA signaling via synthetic agonists provides a reversible, high-precision strategy to optimize wheat water-use efficiency by coupling persistent antitranspirant effects with the metabolic reinforcement of carbohydrate reserves.

**Abstract:**

The phytohormone abscisic acid (ABA) and its PYR/PYL/RCAR receptors are central targets for improving crop drought tolerance. In hexaploid wheat (*Triticum aestivum*), the actual number of ABA receptors (TaPYLs) has been a matter of debate, ranging from 26 to 38. This study refines this classification, demonstrating that 12 putative TaPYLs belong to the Bet v/START/Polyketide cyclase superfamily, but lack key structural features of functional ABA receptors. Indeed, structural and bioinformatic analyses confirm that their ligand-binding cavities cannot accommodate ABA and lack key residues for ABA binding. As a result, whereas TaPYL1, TaPYL4, and TaPYL6 mediate ABA-dependent inhibition of PP2Cs, the TraesCS3B02G160200 putative receptor does not. Therefore, wheat has only 26 potential TaPYL ABA receptors. Given the biotechnological potential of the ABA pathway, we investigated its chemical activation using the synthetic receptor agonist iSB09. This compound is perceived by highly expressed TaPYLs and induces a more persistent antitranspirant effect than exogenous ABA. Plants treated with iSB09 consumed approximately 25% less water over 15 days without incurring a biomass penalty, resulting in significantly higher water productivity. Beyond regulating transpiration, agonist treatment increased fructan and starch levels compared with controls. A short-term RNA-seq experiment revealed that ABA treatment triggers a coordinated reprogramming of carbohydrate metabolism genes, including the upregulation of genes involved in the biosynthesis of protective osmolytes (fructans and raffinose) and starch, coupled with the downregulation of genes involved in starch mobilization. In summary, this work provides a clarified census of the wheat TaPYL family, establishes chemical activation of ABA signaling as an effective strategy to boost water-use efficiency, and suggests that the reinforcement of reserve and protective carbohydrate pathways is a key metabolic mechanism linked to ABA signaling.

**Supplementary Information:**

The online version contains supplementary material available at https://doi.org/10.1007/s00425-026-05216-3.

## Introduction

Water availability is a major limitation for crop productivity. Drought can reduce crop yields by up to 50%; therefore, developing strategies to improve crop yields under limited water supplies is required (Condon et al. 2020). Agriculture accounts for 70% of total water consumption, and global water needs in 2030 are projected to exceed the sustainable water supply by 40% (FAO 2025). Global warming and climate change exacerbate the effects of ordinary seasonal weather variations, constituting a major threat to crop production (FAO 2025). The phytohormone abscisic acid (ABA) plays a crucial role in coping with water deficit, regulating water-use efficiency by controlling stomatal opening and making long-term adjustments to maintain net carbon assimilation (Liu et al. 2025). Plant transpiration through stomata is the major source of water loss during gas exchange for photosynthesis (Dittrich et al. 2019). ABA controls stomatal aperture and modulates plant transpiration, root water uptake, and hydrotropism (Antoni et al. 2013; Dietrich et al. 2017; Dittrich et al. 2019; Miao et al. 2021; Pizzio et al. 2024).

Wheat (*Triticum aestivum*) is a major staple crop, and this plant is sensitive to drought stress. Wheat can be grown in a wide range of agroclimatic environments; however, many of these environments face drought stress as a major productivity challenge. This cereal is cultivated for its high yield, valuable nutritional properties, and unique technological characteristics of the grain. Periodic water deficiency is one of the most critical environmental factors limiting cereal yields, especially during the reproductive period and in spring cereals, which do not use winter soil water reserves. The improvement of wheat for drought tolerance is limited for several reasons (Hura et al. 2020). First, drought may cause dramatic changes in many plant physiological parameters, which need to be measured and understood. Second, the genotype-environment interaction adds further complexity to plant response. Third, drought tolerance is a complex trait controlled by many genes. For example, quantitative trait locus (QTL) mapping has identified numerous genomic regions with both minor and major effects associated with drought tolerance in cereals (Gietler et al. 2020). Interestingly, detected QTLs for agronomic, physiological, and metabolite traits in wheat (and barley) using bi-parental mapping populations support a key role for ABA in plant drought response (Gietler et al. 2020). Wheat accumulates ABA under drought stress, and QTLs associated with ABA action can help elucidate the drought-tolerance mechanism. Seven QTLs were identified for ABA response in wheat under drought stress, with the 5 A QTL having the most significant effect (Iehisa et al. 2014). This QTL coincided with the QTL that also encodes drought tolerance, as predicted by carbon isotope ratio, chlorophyll content, and flag leaf rolling (Iehisa et al. 2014). Likewise, another QTL for drought tolerance associated with ABA signaling has been identified in maize (Avramova et al. 2019).

The reduction of stomatal conductance through genetic strategies has been successfully implemented in wheat, barley, and rice to increase water-use efficiency (Hughes et al. 2017; Dunn et al. 2019; Bertolino et al. 2019). Chemical induction of ABA signaling by small molecules that act as ABA receptor agonists, specifically at specific developmental stages and only under stress, may represent an alternative or complementary approach to genetic strategies (Vaidya et al. 2019; Vaidya and Cutler 2022). We developed a new sulfonamide-based agonist, iSB09, that incorporates an innovation in the sulfonamide linker's arrangement and enhances water-use efficiency in tomato and grapevine (Lozano-Juste et al. 2023; Sanchez-Olvera et al. 2024; Bono et al. 2024). Unlike genetic modification, small-molecule technology provides reversible, dose-dependent control over ABA signaling, promoting positive ABA-mediated responses to mitigate drought stress (Bono et al. 2025). For example, regulation of stomatal closure, wax thickening, antioxidant system activation, compatible osmolyte accumulation, and synthesis of protective proteins (e.g., LEA proteins) (Hura et al. 2020). Additionally, ABA promotes changes in root architecture, activates proteins associated with active water transport, and alters hydraulic conductivity. Taken together, these adaptations enhance yield under drought stress. However, the overaccumulation of ABA or sustained ABA signaling also leads to pollen sterility, reduced grain filling, thermodormancy, accelerated leaf senescence, and decreased photosynthetic efficiency (Hura et al. 2020). Therefore, a balanced tradeoff in ABA signaling is required for optimum yield under drought stress, and non-constitutive approaches can avoid growth penalties under non-stress conditions (Liu et al. 2025).

**Fig. 1:**
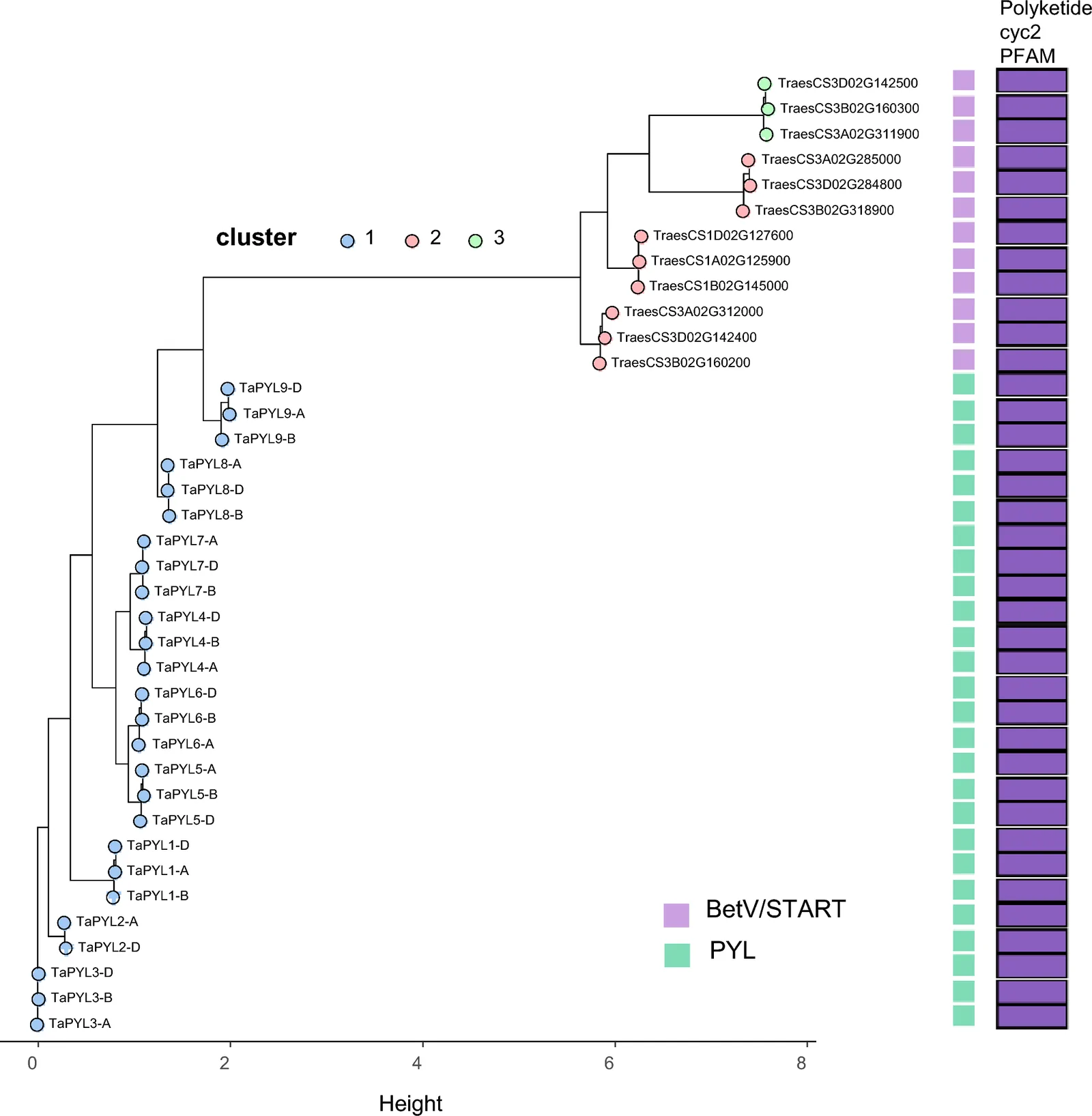
The PYL cluster, containing 26 genes (TaPYL1-TaPYL9 homeologous triplets), diverges from other members of the Polyketide_cyc2 superfamily. Maximum-likelihood phylogenetic tree of 38 bread wheat proteins containing the Polyketide_cyc2 PFAM domain (PF10604), inferred under the WAG+F+G4 substitution model. Based on cophenetic distance clustering, the phylogeny was subdivided into three major clusters. The classification of the 38 proteins into the BetV/START/Polyketide cyclase protein superfamily (BetV/START), including the specific branch of the PYL protein family (green), is indicated on the right

Overexpression of specific wheat ABA receptors (TaPYLs) improves water use efficiency (Mega et al. 2019; Mao et al. 2022). For instance, the overexpression of *TaPYL1-1B* reduces water usage and confers drought tolerance. Candidate gene association analysis revealed that a favorable allele, TaPYL1-1^BIn−442^, confers increased *TaPYL1-1B* expression in Chinese drought-tolerant genotypes (Mao et al. 2022). Overexpression of *TaPYL4* enhances wheat ABA sensitivity, resulting in reduced water consumption and increased water productivity (Mega et al. 2019). At least two different studies have reported the census of *TaPYLs* in the wheat genome (Mega et al. 2019; Lei et al. 2021). Whereas Mega et al. identified 9 groups of homoeologous *TaPYLs* (*TaPYL1* to *TaPYL9*), Lei et al. identified 13 groups (*TaPYL1* to *TaPYL38*). Given the hexaploid nature of the wheat genome, each *TaPYL* represents a homoeologous triplet contributed by subgenomes A, B, and D (except *TaPYL2*, whose sequence is absent in subgenome B); therefore, 26 *TaPYLs* were finally identified by Mega et al. (2019). Compared with Mega et al., the analysis by Lei et al. identified four additional groups of putative *TaPYL* homoeologous triplets, adding 12 putative ABA receptors to the wheat hexaploid genome, bringing the total to 38 TaPYLs (Fig. 1; *TraesCS3D02G142500* to *TraesCS3B02G160200*; Supplementary Fig. 1). We investigated whether the additional putative PYLs identified by Lei et al. (2021) represent bona fide functional ABA receptors, given that they were identified solely through bioinformatic analysis. PYLs belong to the Bet v (*Betula verrucose*)/START (star-related lipid transfer)/Polyketide cyclase protein superfamily, which is an extensive family and includes proteins that bind ABA and qualify as ABA receptors, but also contains different subfamilies that bind other hydrophobic ligands, such as brassinosteroid hormones, lipids, antibiotics, and precursors of the lacrimatory factor (Radauer et al. 2008; Iyer et al. 2001; Silvaroli et al. 2017). To distinguish actual PYLs from other wheat proteins lacking ABA receptor function, we performed bioinformatic and structural analyses, focusing on key conserved amino acid residues critical for ABA receptor activity. Additionally, we investigated activation of TaPYL-mediated ABA signaling by the ABA receptor agonist iSB09, its effect on wheat physiological responses, and whether chemical induction of ABA signaling by agonists may represent an alternative approach to regulating water-use efficiency and water productivity.

## Materials and methods

### UPGMA tree construction

A phylogenetic tree was constructed using the Unweighted Pair Group Method with Arithmetic Mean (UPGMA) in MEGA11 (version 11.0.13; Tamura et al. 2021). Amino acid sequences from the 38 proteins reported by Lei et al. (2019) were aligned using ClustalW with default parameters. Evolutionary distances were estimated using the Poisson substitution model with uniform rates among sites. Gaps and missing data were handled using the pairwise deletion method. Statistical support for the tree topology was assessed using the bootstrap method with 100 replicates. The resulting consensus tree was visualized using MEGA11 under homogeneous lineage patterns.

### Sequence and structure-based alignment

Multiple sequence alignment of wheat ABA receptors (Mega et al. 2019) was performed using ClustalW with default parameters. The alignment was visualized and annotated using ESPript 3.0 (Robert and Gouet 2014; https://espript.ibcp.fr), which allows graphical representation of conserved residues and secondary-structure elements. The secondary structure of *Arabidopsis thaliana* PYL9/RCAR1 (AtPYL9) was used as a reference and extracted from its crystal structure (PDB ID: 3W9R; Nakagawa et al. 2014). Conserved residues are colored according to sequence identity, and functionally relevant motifs, such as the “gate” and “latch” regions, are highlighted based on structural annotation.

### Expression and purification of TaPYL receptors, TraesCS3B02G160200 and phosphatases

The wheat ABA receptors *TaPYL1* (TraesCS1B02G206600), *TaPYL4* (TraesCS2D02G087500), *TaPYL6* (TraesCS1D02G293600), and *TaPYL8* (TraesCS1B02G145800), as well as *ΔNAtHAB1* (179–511) and *ΔNTaPP2C4* (81–397) (TraesCS3B02G394600) phosphatases, were cloned into the pETM11 (Novagen) expression vector and constructs were introduced into *E. coli* BL21 (DE3) cells. A representative member, i.e., TraesCS3B02G160200, of the PF10604 PFAM motif (Polyketide_cyc2) was also cloned into pETM11, and the construct was introduced into *E. coli* BL21 (DE3) cells. Transformed colonies were grown overnight in LB medium supplemented with kanamycin (100 µg/mL) at 37 °C with shaking until saturation. For protein expression, cultures were inoculated at a 1:100 dilution into fresh LB medium containing the same kanamycin concentration. When cultures reached an optimal optical density at 600 nm between 0.6–0.7, protein expression was induced by adding 1 mM IPTG, and the incubation continued overnight at 16 °C. Cells were collected by centrifugation at 5000 × g for 20 min at 4 °C.

*E. coli* expressing TaPYLs, TraesCS3B02G160200, and phosphatases were purified using Ni^2^⁺-NTA affinity chromatography columns (Qiagen). Cell pellets were resuspended in lysis buffer containing 50 mM Tris–HCl, pH 8.0, 250 mM KCl, 0.1% Tween-20, 10 mM β-mercaptoethanol, 10% (v/v) glycerol, and 10 mM imidazole. Cells were lysed by sonication, and lysates were clarified by centrifugation at 13,000 g for 30 min at 4 °C. The supernatant was loaded onto the Ni^2^⁺ resin column and washed with a buffer of similar composition, but containing 20% glycerol and 30 mM imidazole. Proteins were eluted using the same buffer supplemented with 250 mM imidazole and stored at − 80 °C in the presence of 1 mM DTT.

### Phosphatase inhibition assays

Protein integrity was verified by SDS–polyacrylamide gel electrophoresis (SDS–PAGE), followed by InstantBlue staining (Expedeon). Phosphatase activity was assayed using 25 mM p-nitrophenyl phosphate (pNPP) as substrate in the presence of 10 mM MgCl₂, 1 μM PP2C (*ΔNAtHAB1* or *ΔNTaPP2C4*), and 4 μM of the indicated receptors. Proteins were pre-incubated with ligands for 30 min at room temperature before the addition of the substrate. The reaction was initiated by adding pNPP, and absorbance at 405 nm was monitored over 20 min using a VICTOR X5 plate reader (PerkinElmer). All assays were performed in duplicate.

### Plant material and experimental setup

Bread wheat (*Triticum aestivum* L. var. MPB-Bobwhite26, hereafter referred to as ‘Bobwhite’) was used in this study. Seeds were germinated on wet filter paper at 4 °C in the dark for three days. Three-day-old seedlings were then transferred to 500 mL pots (one seedling per pot) filled with 300 g of substrate (Vithal Garden fertiliser 10 in 1; NPK 5–4-3 + Mg + 72% Organic matter + Bioestimulant Nitrosphere; an additional 1.9 g of NPK 5–4-3 fertilizer (Vithal garden^®^, 71–76% organic matter, 24–44% organic C, substrate class A) was added to each pot to ensure proper nutrient availability.

A separate set of pots was used to determine soil field capacity by watering to full capacity, allowing for leaching, and then drying completely. The 20 potted plants were placed in a growth chamber under the following conditions: a 16 h light/8 h dark photoperiod, day/night temperatures of 25 °C/15 °C, photosynthetically active radiation (PAR) of 400 µmol m⁻^2^ s⁻^1^, and a relative humidity of 40%. Plants were regularly watered with tap water and rotated within the chamber to minimize edge effects.

Treatment and phenotyping of the plants began at 34 days (d), and we defined this time point as 0 days (see the scheme in Fig. 5A). Before treatments, water field capacity and stomatal conductance were recorded. The whole plants (including adaxial and abaxial leaf surfaces) were sprayed with either 100 µM ABA agonist iSB09 (in a water solution containing 0.05% v/v Tween-20, and 0.1% v/v DMSO) or a mock solution (water with 0.05% v/v Tween-20 and 0.1% v/v DMSO). We established initially two groups of 10 plants each, and phenotypic measurements were taken according to the following program: field capacity was recorded on days 0, 1, 3, 7, 9, 11, and 15 after treatment; porometer (LI-600) readings were taken on days 0, 1, 3, and 7; gas exchange measurements were obtained on day 7; and biomass was evaluated on days 7 and 15. On day 7, five plants per treatment group (*n* = 5 biological replicates) were sampled and immediately frozen in liquid nitrogen for the analysis of soluble sugars and starch. From day 7 onwards, the remaining 5 plants per group were used for the subsequent phenotypic measurements. Following the final measurements on day 15, the remaining 5 plants per treatment were also frozen for sample analysis.

### Sampling and biochemical analyses

At 7 d and 15 d, five plants per group and time point were sampled using liquid nitrogen, and all leaves from each plant were collected as a single pool, excluding the stems, and stored at − 80 °C for further biochemical analyses. After ethanol/water extraction according to Morcuende et al. (2004), the soluble sugars glucose, fructose, sucrose, and fructans were determined using a spectrophotometric assay coupled to NADP reduction. Total soluble sugars (TSS) were calculated as the sum of these sugars and expressed as hexose equivalents. Moreover, in the ethanol/water-extraction pellet, starch was determined as described by Morcuende et al. (2004).

### Thermography images

*T. aestivum* plants were grown in a controlled environment chamber at 26 °C under a 16-h light/8-h dark photoperiod. Four-week-old plants were sprayed with 10 mM MES (pH 5.7) + 0.02% Silwet L-77, supplemented with the indicated ligand concentration, or 0.1% DMSO as a mock-treated control. Infrared (IR) images of plants were acquired 4 h after treatment using a FLIR E95 thermal camera. Quantification was performed using FLIR Tools software, with 10 sections per plant analyzed. At least six plants per treatment were used, and experiments were repeated twice. The mean leaf temperature and its standard deviation were calculated for each treatment.

### Stomatal conductance analyses and measurements of photosynthetic rate

Stomatal conductance (gs) was measured on fully developed leaves using a portable LI-600PF porometer and fluorometer (LI-COR, Lincoln, NE, USA) at ambient conditions and a PAR of 150 µmol m⁻^2^ s⁻^1^. Measurements were performed on approximately 5 leaves per plant, using 6 plants per treatment, from the same plants used for thermal imaging. Gas-exchange measurements on fully developed leaves were taken around midday with an infra-red gas analyzer CIRAS-3 (PP Systems, USA) with the following conditions: 420 µmol CO_2_ mol^−1^, 1400 µmol m-2 s-1 of light intensity, 25 °C of cuvette temperature, 40% of relative humidity, 300 mL min^−1^ of flow rate, and 2.19 ± 0.39 kPa of vapor pressure deficit. In addition to the photosynthetic parameters, instantaneous (WUE) and intrinsic (iWUE) water-use efficiencies were calculated as the ratios of photosynthetic rate to transpiration (E) or to gs, respectively.

### RNA-seq analysis

The RNA-seq data discussed in this publication have been deposited in NCBI’s Gene Expression Omnibus (GEO) under the GEO Series accession GSE317722. Wheat seeds (Bobwhite) were germinated in glass flasks (*n* = 12 seeds per flask, with three flasks per treatment) containing MS medium without sucrose. Following 7 days of growth at 23 °C (16 h light, 8 h dark), the remaining medium was removed and replaced with MS medium without sucrose, supplemented with 20 µM ABA or 0.1% DMSO for the mock treatment. The plant material was collected 4 h post-treatment. The RNA extraction of plant material was performed using the SpectrumTM Plant Total RNA kit (Sigma-Aldrich). The quality of the samples was verified using an Agilent 2100 Bioanalyzer. cDNA synthesis and sequencing of cDNA libraries were performed using DNBSEQ technology at the Beijing Genomics Institute, producing at least 20 million clean paired-end 100-bp reads per sample. The Bioinformatics core facility at the IBMCP carried out RNA-seq data analysis. In brief, after quality analysis of raw reads with FastQC (https://www.bioinformatics.babraham.ac.uk/projects/fastqc/) and quality trimming and adapter removal of raw reads with cutadapt (http://journal.embnet.org/index.php/embnetjournal/article/view/200), clean read pairs longer than 20 nt were aligned to the International Wheat Genome Sequencing Consortium (IWGSC) RefSeqv1.1 (https://www.wheatgenome.org/) using kallisto (Bray et al. 2015; Borrill et al. 2016; Ramirez-Gonzalez et al. 2018) with default parameters for transcript/isoform quantification. Differential expression analysis of the transcripts was performed using DESeq2. Genes with an absolute value of log2FC > 1 and *p*-adjusted value (padj) < 0.05 were considered differentially expressed genes (DEGs).

### Real-time qPCR analysis

Seven-day-old wheat seedlings (cv Bobwhite) were treated with mock or 20 μM iSB09 for 4 h. Total RNA was extracted using a NucleoSpin RNA plant kit. cDNA was synthesized from 1 μg of purified RNA using 30 U of RevertAid Reverse Transcriptase (Thermo Scientific). Real-time quantitative PCR (RT-qPCR) was performed using PyroTaq EvaGreen qPCR Master Mix 5X from Cultek, which includes EvaGreen® Dye and carboxy-X-rhodamine (ROX) as a passive reference dye. The primer pairs used for the quantitative analysis of wheat genes are: 5’-AGGAGAGCTCTGACGATGACCG and 5’-GCCGGATCGTAGAACGTCTTGG for *TraesCS6D02G026400* (fructan biosynthesis) and 5’-GCGGAGAACGAGCCCAACATGA and 5’-GCGTGGAGCTCGTCGTAGAAGC for *TraesCS7A02G134100* (raffinose biosynthesis). RT-qPCR was performed on a QuantStudio 3 (Applied Biosystems). Relative quantification of gene expression was performed using the 2^-ΔΔCT (comparative CT) method. Expression was normalized to values obtained for cyclophilin (*TraesCS7A02G410100*) (Bocian et al. 2025). The mean and standard deviation of 3 independent samples are shown.

### Statistical analysis

Statistical analyses were performed using GraphPad Prism v8.0. For simple comparisons between two experimental groups (e.g., mock vs. agonist treatment), data were analyzed using a two-tailed Student’s *t*-test. For multi-group comparisons involving three or more experimental conditions, statistical significance was assessed using a one-way analysis of variance (ANOVA) followed by Tukey's post-hoc test. Data are expressed as the mean ± standard deviation (SD), and differences were considered statistically significant at *p* < 0.05 (*).

## Results

### Updated census of the gene family encoding ABA receptors in *Triticum aestivum*

Genome-wide searching of the PF10604 PFAM motif (Polyketide_cyc2) identified 38 PFAM-containing proteins in the bread wheat reference genome (v1.1) (Fig. 1). Among these 38 proteins, 26 were designated as TaPYL proteins by Mega et al. (2019), while the remaining 12 were classified as additional members of the BetV/START/Polyketide cyclase protein superfamily. To assess the sequence relatedness of the 38 proteins, a phylogenetic analysis was performed. Protein sequences were first aligned using MAFFT with the localpair and maxiterate parameters set to 1000, resulting in a high-quality multiple sequence alignment. The aligned sequences were then used to infer a maximum-likelihood phylogeny with IQTREE3 using the model selection option -m MFP, 1000 ultrafast bootstrap replicates (-B 1000), 1000 SH-aLRT tests, and –bnni optimization to reduce bias. Clustering of the proteins based on the cophenetic distances of the resulting tree, i.e., a measure of how faithfully a dendrogram preserves the pairwise distances, revealed that canonical TaPYL1 to TaPYL9 (Mega et al. 2019) grouped in cluster 1. In contrast, the putative 12 TaPYLs included by Lei et al. (2021) were grouped in clusters 2 and 3 (Fig. 1). Therefore, these results suggest that the putative 12 TaPYLs are related, but distant to canonical TaPYLs.

**Fig. 2:**
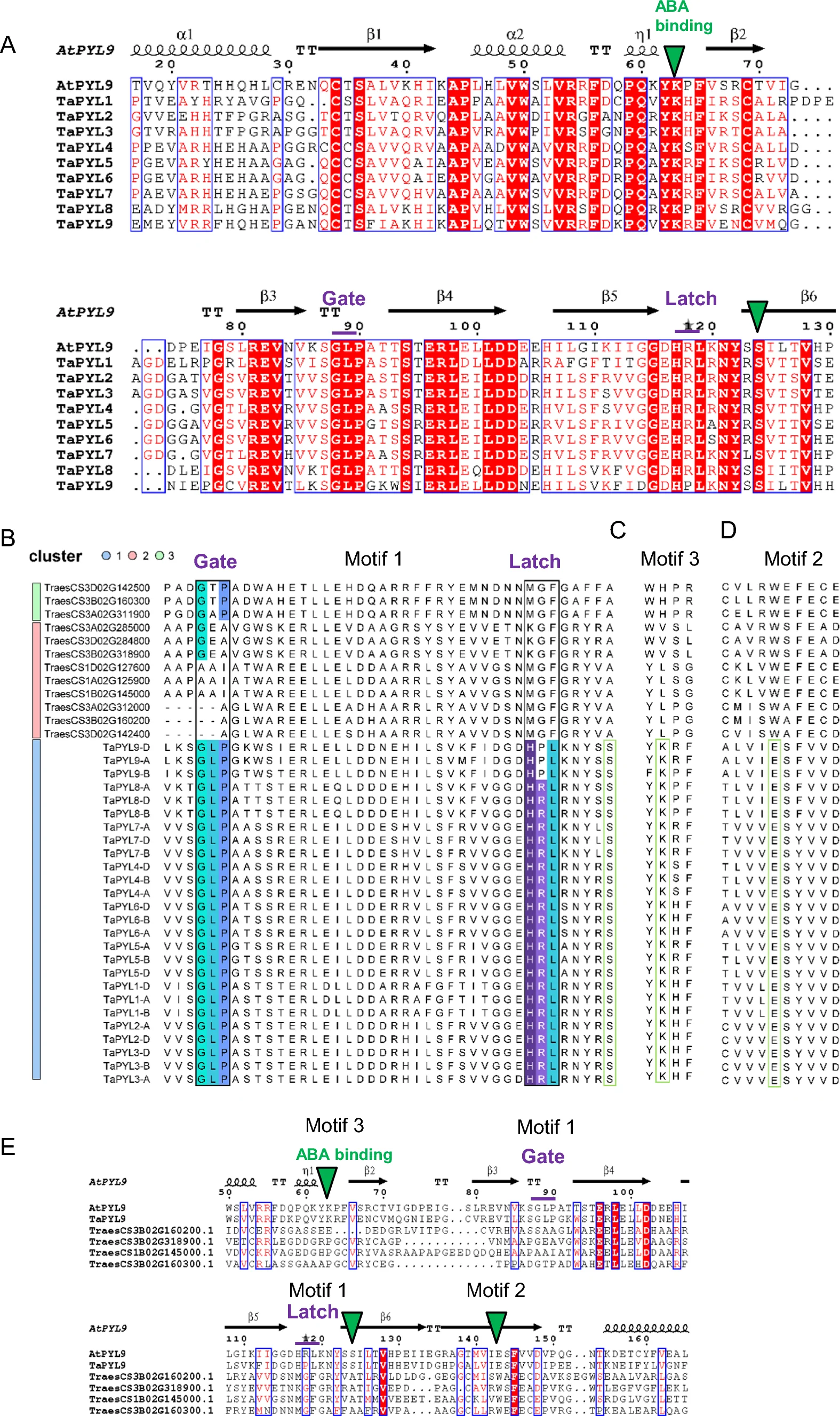
Amino acid alignment reveals the lack of key residues involved in ABA binding in 12 wheat proteins previously identified as putative TaPYLs. **A** Amino acid sequence and secondary structure alignment of canonical TaPYLs with AtPYL9. The figure was generated with ESPript 3.2 (https://espript.ibcp.fr/ESPript/ESPript/). The PDB code for the ABA-bound form of AtPYL9 is 3W9R. Key residues for gate and latch loops of ABA receptors and ABA binding are conserved in all TaPYLs, except for a change in the latch loop of TaPYL9. **B**, **C**, **D** Multiple sequence alignment of the 38 wheat Polyketide_cyc2 PFAM-containing proteins reveals the lack of key residues for ABA binding in 12 putative TaPYLs. Multiple sequence alignment of regions corresponding to (**B**) motif 1, **C** motif 3, and **D** motif 2 identified by the MEME suite across the 38 Polyketide_cyc2 PFAM-containing proteins. Proteins are grouped into three major clusters based on phylogenetic analysis. In the motif 1 region (**B**), the Gate and Latch elements are indicated, and sequence discrepancies among proteins are highlighted. Residues involved in ABA binding by PYL proteins are also highlighted within the alignments (**B**–**D**). **E** Summary of the differences in key regions for ABA binding between the putative TaPYLs and the actual AtPYL9 ABA receptor. TaPYL9 shows a change in the latch gate that might affect its capacity to bind ABA

We performed sequence alignment of the TaPYL1-TaPYL9 receptors identified by Mega et al. (2019), including AtPYL9, to guide structural alignment using ESPRIT software, which revealed the conservation of amino acid sequence hallmarks that define an ABA receptor (Fig. 2A). For example, invariant residues in the gate (GLP) and latch (HRL) loops were identified in all the receptors, except TaPYL9, which bears a change (HPL) in the latch loop (Fig. 2A). We performed a similar alignment with the four additional groups of putative TaPYL homoeologous triplets (Lei et al. 2021) (Fig. 2B). Notably, the putative 12 TaPYLs lack key invariant residues in the gate and latch loops of bona fide ABA receptors, which suggests that these proteins are not functional ABA receptors (Fig. 2B; Supplementary Fig. 1). Moreover, key residues for ABA binding are lacking, such as the conserved Lys residue (motif 3), which is located at the bottom of the ligand-binding cavity of canonical PYL ABA receptors (Santiago et al. 2009) (Fig. 2C). This Lys residue establishes a direct polar contact between its amine group and the ABA’s carboxylate group, and mutations in this residue abolish ABA binding (Santiago et al. 2009; Park et al., 2015). Additionally, other conserved residues in ABA receptors, such as those equivalent to Glu143 (motif 2) and Ser124 (close to the latch) of AtPYL9, which establish hydrogen bonds mediated by water molecules with the ABA’s carboxylate group, are lacking in the 12 proteins (Fig. 2B and D). Thus, the sequence alignment data presented in Fig. 2B–E suggest that the putative 12 TaPYLs do not qualify as ABA receptors.

### Structural analysis reveals that 12 putative TaPYLs belong to the polyketide cyclase protein superfamily, but are not bona fide ABA receptors

To further investigate the function of the proteins encoded by the 12 putative *TaPYL* genes (TraesCS3D02G142500 to TraesCS3D02G142400), we performed a protein BLAST (BLASTp) search using the sequences of representative members, namely TraesCS3B02G160200, TraesCS3B02G318900, TraesCS1B02G145000, and TraesCS3B02G160300. As a result, we identified homology to lachrymatory factor synthase (LFS)-like proteins (Supplementary Fig. 2). Indeed, both TraesCS3B02G318900 and TraesCS3B02G160300 are annotated as LFS in the *Triticum aestivum* genome (https://blast.ncbi.nlm.nih.gov/Blast.cgi). The LFS functions as an onion-like defense mechanism against microbes and animals; however, the existence of such a pathway in wheat is unlikely. The LFS of *Allium cepa* shows low sequence similarity to PYL-like ABA receptors; however, it belongs to the Bet v/START/Polyketide cyclase protein superfamily (Silvaroli et al. 2017). This protein superfamily comprises the PYLs and numerous other proteins that share structural similarity, but exhibit different ligand specificities (Gajhede et al. 1996; Markovic-Housley et al. 2003; Radauer et al. 2008). The Bet v scaffold diversified during evolution into different families that exhibit low sequence similarity, but maintain a common helix-grip fold for binding hydrophobic ligands (Radauer et al. 2008).

**Fig. 3:**
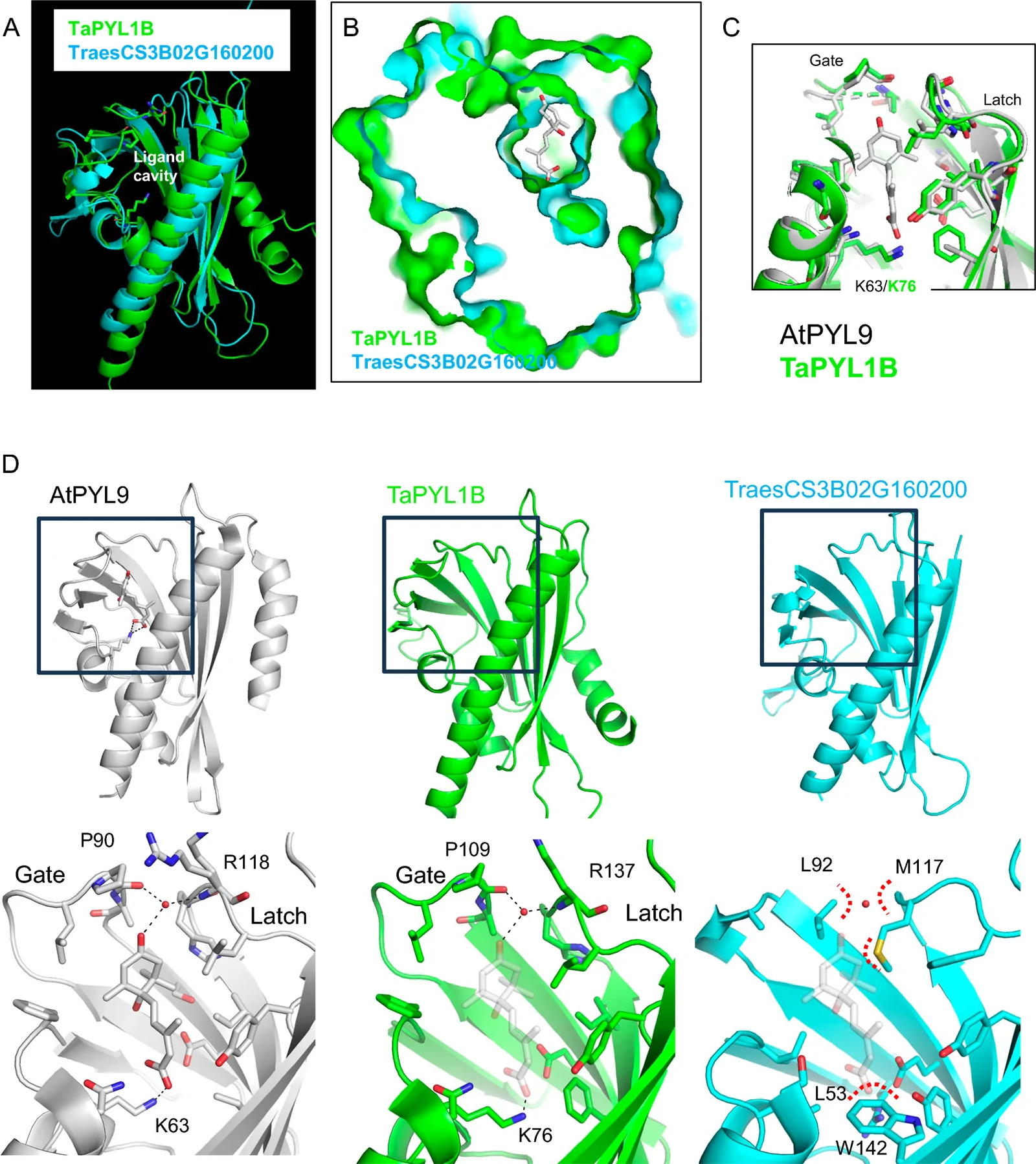
Structural analysis of TraesCS3B02G160200 compared to the bona fide ABA receptors AtPYL9 and TaPYL1B reveals that the predicted binding cavity of TraesCS3B02G160200 cannot accommodate the ABA molecule. **A** The predicted structures of TaPYL1B (green) and TraesCS3B02G160200 (blue) display the Bet v fold, forming a ligand-binding cavity. **B** A section of a surface representation of the ligand-binding cavity in the predicted structures of TaPYL1B and TraesCS3B02G160200. While ABA fits into the cavity of TaPYL1B, it protrudes into the ligand-binding cavity of TraesCS3B02G160200m, thereby hindering ABA binding. **C** A detail of the binding pocket of TaPYL1B and AtPYL9 highlights those residues involved in ABA binding, specifically K63/K76 for coordination with the ABA’s carboxylate group. **D** A section of the ABA-binding pocket in the experimental structure of the AtPYL9-ABA complex (left), and in the predicted structures of TaPYL1B (center) and TraesCS3B02G160200 (right). Side chains involved in ABA binding and in the formation of the Trp-lock are shown. ABA and the water molecule participating in the Trp-lock were also modeled in the predicted structures. Red dashed lines indicate predicted close contacts that lead to steric hindrance between ABA and the modeled TraesCS3B02G160200 structure

In onion, using the PYL10 structure as a representative of the Bet v/START/Polyketide cyclase protein superfamily, structural studies have elucidated the catalytic activity of LFS on the (*E*)− 1-propenesulfenic acid substrate (Silvaroli et al. 2017). In our case, we have chosen TraesCS3B02G160200 as a representative of the 12 putative TaPYLs and TaPYL1B as a representative of the canonical TaPYLs. To compare the binding cavities of TaPYL1B and TraesCS3B02G160200, we predicted their structures using AlphaFold3 (Abramson et al. 2024) (Fig. 3A). As expected, both predicted proteins display the characteristic Bet v fold, consisting of a curved seven-stranded β-sheet wrapped around a long C-terminal α-helix, which forms a hydrophobic cavity for ligand binding (Fig. 3A). Whereas the ligand binding cavity of TaPYL1B can accommodate the ABA molecule, TraesCS3B02G160200 cannot (Fig. 3B; Supplementary Fig. 3A and B). Indeed, substantial differences between TaPYL1B and TraesCS3B02G160200 emerged in key areas for ABA binding (gate, latch, conserved Lys) when comparing the AlphaFold3 predicted models with the experimentally determined structure of ABA-bound AtPYL9 (Nakagawa et al. 2014) (Fig. 3C; Supplementary Fig. 3C). Overlaying the predicted binding cavities reveals that only TaPYL1B aligns with that of AtPYL9 and conserves most of the polar and hydrophobic residues essential for ABA binding (Fig. 3C). Moreover, AtPYL9 and TaPYL1B are predicted to adopt the canonical ABA receptor conformation and to form the ternary ABA-PP2C complex, thereby supporting their function as ABA-dependent PP2C inhibitors (Fig. 3D, left and middle panels). Thus, TaPYL1B displays proper location of the Pro109 and Arg137 residues to coordinate the water molecule required to form the Trp lock linking the receptor, ABA, and the phosphatase (Melcher et al. 2009). Additionally, Lys76 is located at the bottom of the ligand-binding cavity to coordinate with the ABA carboxylate (Fig. 3D, middle panel). Conversely, the TraesCS3B02G160200 model lacks the gate-and-latch loop architecture required for PP2C interaction (Fig. 3D, right panel). Moreover, some residues in the ligand-binding cavity would impose steric hindrance to ABA or to the water molecule that assembles the Trp lock, for example, Met117, Leu92, and Trp142. Finally, TraesCS3B02G160200 lacks the conserved Lys residue required to establish polar contact with ABA’s carboxylate; instead, Leu53 occupies an equivalent position (Fig. 3D, right panel).

The above structural studies indicate that the predicted binding cavity for TraesCS3B02G160200 cannot accommodate the ABA molecule, unlike that for TaPYL1B. Given the phylogenetic analysis described in Fig. 1, we suggest that other members of clusters 2 and 3, which contain the four additional homeologous triplets of putative TaPYLs identified by Lei et al. (2021), are not bona fide ABA receptors, but instead represent additional members of the Bet v/START protein superfamily whose ligand is still unknown.

### Regulation of wheat transpiration by iSB09 enhances water productivity

**Fig. 4:**
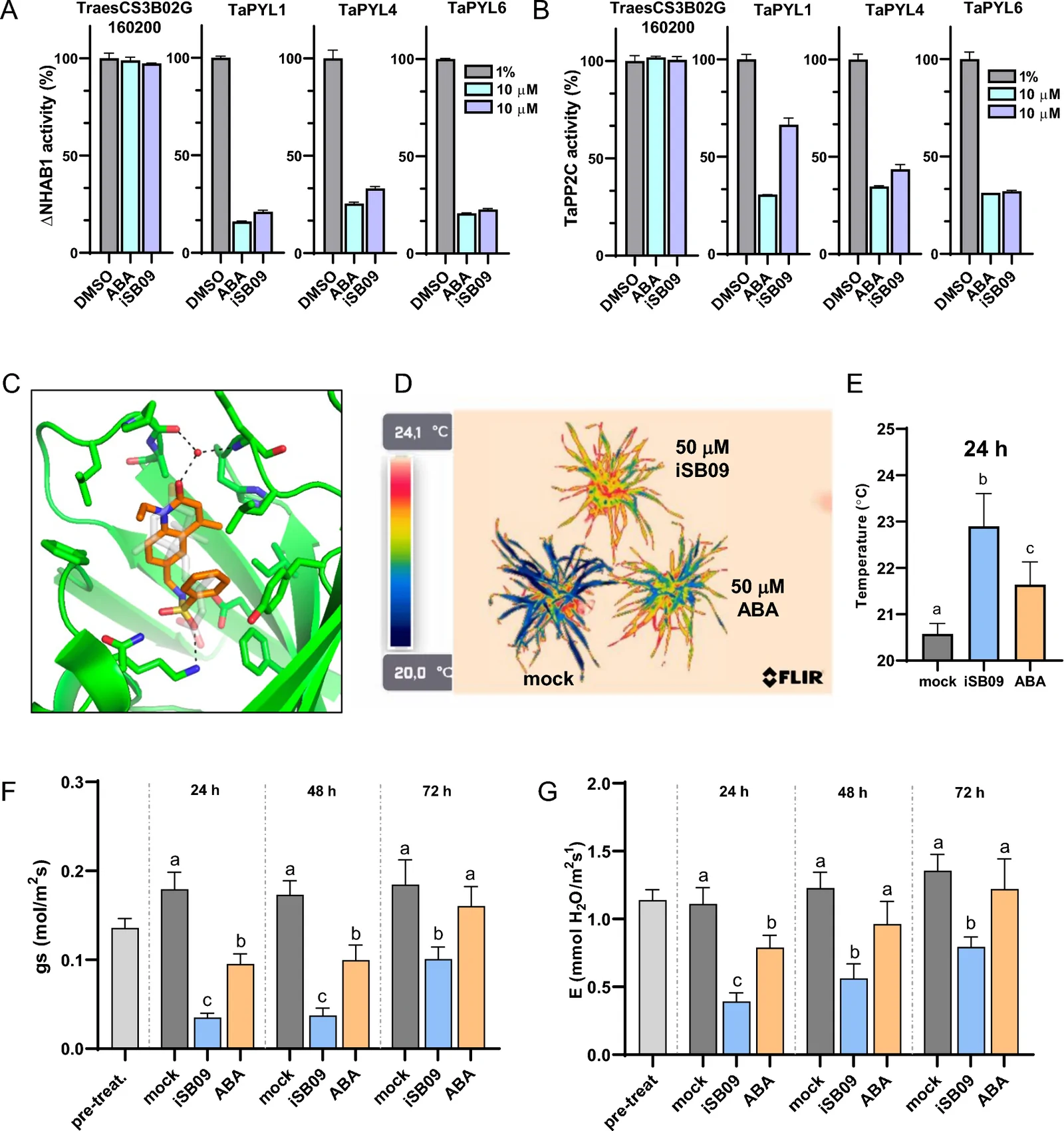
Activation of ABA signaling by iSB09 results in more efficient regulation of transpiration than exogenous ABA treatment. **A**, **B** PP2C inhibition assays reveal that iSB09 activates the ABA receptors TaPYL1, TaPYL4, and TaPYL6. In vitro PP2C assays in which the indicated TaPYLs were incubated with ΔN-HAB1 PP2C (panel A) or TaPP2C4 (panel B) in the absence (no drug, 0.1% DMSO) or presence of 10 μM ABA or iSB09. The TraesCS3B02G160200 protein was unable to inhibit PP2C activity, in contrast to bona fide ABA receptors TaPYL1, TaPYL4, and TaPYL6. **C** Detailed section of the iSB09 binding site indicating the interactions with the conserved Lys and the Trp lock of TaPYL1. **D** Representative infrared images of wheat plants acquired 24 h after treatment with 50 µM iSB09, 50 µM ABA, or mock (0.1% DMSO). The color scale indicates a temperature range of 20 to 24.1 °C. **E** Quantification of plant temperature using FLIR Thermal Studio software. Bars represent the mean ± SD of 5 measurements per plant (*N* = 3). Different letters indicate statistically significant differences (*P* < 0.05). **F**, **G** Quantification of stomatal conductance (gs, panel F) and transpiration rate (panel G) at 24, 48, and 72 h post-treatment of plants treated with mock solution, 50 µM iSB09, or 50 µM ABA. “Pre-treat.” refers to the physiological parameters measured before chemical application

Given the biotechnological importance of actual TaPYLs for drought tolerance, we have explored the use of ABA receptor agonists to activate the ABA pathway on demand. According to gene expression data published by Mega et al. (2019), the most expressed TaPYLs at different developmental stages in “Chinese Spring” *T. aestivum* are TaPYL1, TaPYL4, TaPYL6 (imbibed seed and mature spike), and, to a lesser extent, TaPYL8. We expressed and purified 6-His-tagged TaPYL1, TaPYL4, and TaPYL6, and confirmed their capability to inhibit, in an ABA-dependent manner, the PP2C activity of ΔNTaPP2C4 and ΔNAtHAB1 (Fig. 4A and B). We have recently developed an ABA receptor agonist, termed iSB09, that activates ABA receptors in crops, such as tomato and grapevine, thereby enhancing water use efficiency (WUE) by activating the ABA pathway (Lozano-Juste et al. 2023; Sanchez-Olvera et al. 2024; Bono et al. 2024). Firstly, we tested iSB09’s ability to activate TaPYL1, TaPYL4, and TaPYL6 in PP2C inhibition assays. As a result, we found that iSB09 activated wheat ABA receptors and inhibited both TaPP2C4 and AtHAB1 phosphatases (Fig. 4A and B). We also tested the putative biochemical activity of TraesCS3B02G160200 as an ABA receptor (Fig. 4A and B). Consistent with structural and bioinformatic analyses, TraesCS3B02G160200 failed to inhibit PP2C phosphatases in response to ABA or iSB09 (Fig. 4A and B). Finally, to gain further insights into receptor activation by iSB09, we took advantage of the crystal structure of CsPYL1-iSB09-HAB1 complex (Lozano-Juste et al. 2023). The overall fold of this structure was used to model the iSB09 compound into the ligand-binding pocket of TaPYL1 bound to ABA using the AlphaFold3 model described above (Fig. 4C). The model conserves two major features for ligand binding: the hydrogen bond network that constitutes the Trp lock (Melcher et al. 2009; Rodriguez and Albert 2026), and the hydrogen bond between the SO2 moiety of iSB09 and the conserved Lys76 residue at the bottom of the ligand-binding cavity (Fig. 4C).

Few physiological data are available for the use of ABA receptor agonists in wheat (Vaidya et al. 2019; Roeder et al. 2023). For example, Roeder et al. demonstrated that treatment with 100 μM cyanocyclopropyl analogs (CCPs) reduced leaf transpiration in wheat, as measured by IR thermography 24 h after foliar application. Vaidya et al. found that applying 100 μM opabactin (OPA) resulted in a sustained increase in leaf temperature 5 days after foliar application. However, physiological measurements of stomatal conductance (gs), transpiration (E), or WUE were not reported in previous assays. Using 50 μM iSB09, we also detected an increase in leaf temperature by IR thermography (Fig. 4D and E). Interestingly, this increase was greater in iSB09-treated plants than in ABA-treated plants, suggesting that iSB09 has a significant antitranspirant effect. We compared gs and E in iSB09-treated versus ABA-treated plants grown in growth chambers under controlled conditions. We found that iSB09 diminished both gs and E, and its effect lasted at least 72 h after application (Fig. 4F and G). In contrast, the antitranspirant effect of ABA was reduced relative to iSB09 and did not differ significantly from that of mock-treated plants at 72 h after application.

**Fig. 5:**
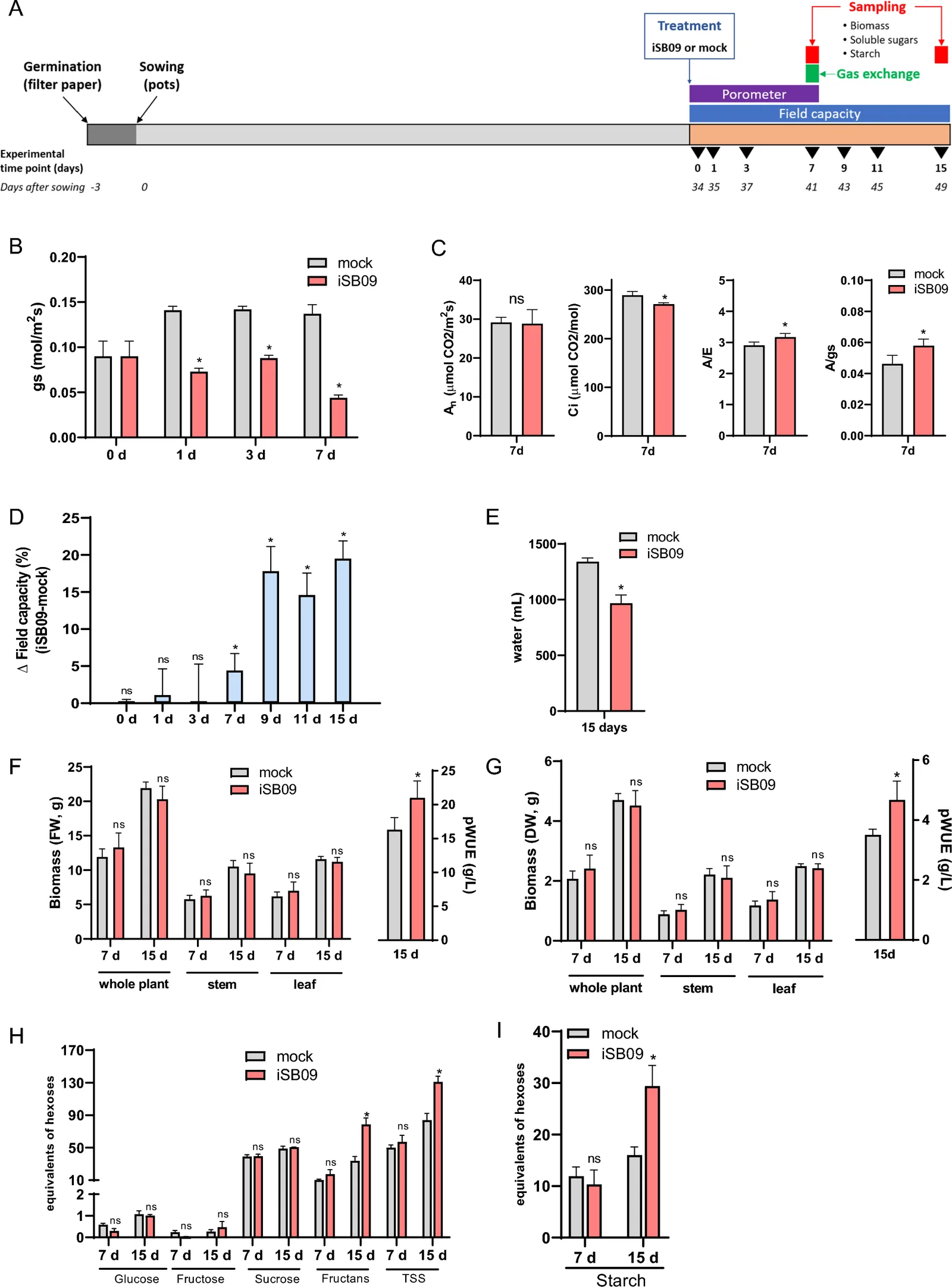
Reduced water consumption, enhanced water productivity, fructan and starch biosynthesis upon treatment with iSB09. **A** Experimental design and timeline for the phenotyping of wheat plants. Plants were treated with either a mock solution or 100 µM iSB09 at 34 days (d) after sowing (defined as 0 days in the experimental time point). Phenotyping analysis (porometer/fluorometer, gas exchange, and biomass) was performed over the 15 d of the experiment. Sampling for biochemical analysis was performed at 7 d and 15 d. **B** iSB09-treated plants show a persistent reduction in stomatal conductance (gs) compared to mock-treated plants. The gs was measured at 0, 1, 3, and 7 days after treatment using a LI-600 porometer. **C** Photosynthetic rate, Ci, WUE, and iWUE measurements using an infrared gas analyzer CIRAS-3 at 7 days after agonist treatment. **D** Increased water field capacity in iSB09-treated plants compared to mock-treated plants. Soil water content was measured over a 15-day period for mock-treated or iSB09-treated plants. The difference (Δ) in field capacity between iSB09-treated and mock-treated plants is shown. **E** iSB09 treatment reduces water consumption over a 15-day period compared to mock-treated plants. Total cumulative water consumed per plant over the 15-day experimental period was monitored. **F**, **G** Higher productivity water-use efficiency (pWUE) is achieved in iSB09-treated plants than in mock-treated plants, without a biomass penalty. Biomass production was monitored by measuring fresh weight (FW, panel E) and dry weight (DW, panel F) of aboveground biomass (stems and leaves) at 7- and 15-day post-treatment. pWUE was calculated as the ratio of biomass produced per unit of water consumed (g/L). **H** Quantification of soluble sugars (glucose, fructose, sucrose, and fructans) and total soluble sugars (TSS) at 7 and 15 days after treatment. Fructan levels were significantly higher (2-fold) in iSB09-treated plants at 15 days. **I** iSB09 treatment leads to a 1.8-fold increase in starch accumulation by the end of the experiment. Starch content was measured at 7 and 15 days after treatment. In panels, the bars represent the mean value of at least five biological replicates, and error bars indicate the standard deviation (SD). Asterisks (*) denote statistically significant differences compared to the mock treatment (*P* < 0.05) using Student's t-test for each time point, comparing the iSB09 with the corresponding mock data

Next, we conducted a longer experiment to assess the persistence of the agonist effect on gs and, ultimately, on soil water consumption at 15 days after treatment (Fig. 5A–C). iSB09 diminished gs even at 7 days after treatment (Fig. 5B). WUE and iWUE were higher at 7 days after agonist treatment, while photosynthesis was not diminished by agonist treatment in spite of reduced intercellular CO_2_ concentration (Ci, Fig. 5C). In addition to Ci, other factors influence the photosynthetic rate, including mesophyll conductance and leaf water potential (Yang et al. 2016; Liu et al. 2025). Results in Fig. 5B suggest that the antitranspirational effect of iSB09 can improve leaf water status and compensate for the reduction in photosynthetic rate (Liu et al. 2025). Given the agonist's efficient control of water consumption, water field capacity was higher in iSB09-treated plants than in mock-treated plants (Fig. 5D). Additionally, we measured water consumption and biomass production (Fig. 5E–G). Interestingly, iSB09-treated plants consumed 28% less water than mock-treated plants at 15 days after treatment (Fig. 5E). Biomass production, either fresh weight (FW) or dry weight (DW), was not significantly different in mock- and iSB09-treated plants (Fig. 5F and G). As a result, the water use efficiency, defined as water productivity, i.e., the quantity of biomass generated per unit of water consumed, was approximately 20–25% higher in iSB09-treated plants at 15 days after treatment (Fig. 5F and G). These results suggest that agonist treatment reduces water consumption without compromising photosynthetic rates and biomass production, thereby increasing water productivity.

### Activation of ABA signaling by agonist treatment increases the levels of fructans and starch

Carbohydrate metabolism is a central pathway in cereal studies for its influence on grain yield and stress responses. Therefore, we measured sugar content 15 days after treatment and found that glucose, fructose, and sucrose levels did not differ significantly between mock- and iSB09-treated plants (Fig. 5H). However, the fructan content was considerably higher in agonist-treated plants (Fig. 5H). Fructans, complex fructose polymers, have previously been reported to protect against abiotic stress in other species, for example, by stabilizing membranes during drying or freezing (Livingston et al. 2009), and have been associated with increased drought and freezing tolerance (Livingston et al. 2009; Shimosaka and Ozawa 2015). While our study did not directly test drought or freezing tolerance, the increased fructan accumulation observed here is consistent with activation of this protective pathway. Fructan content at 15 days after treatment was 2-fold higher in agonist-treated than in mock-treated plants, which also resulted in increased levels of total soluble sugars (TSS) at the end of the experiment (Fig. 5H). Interestingly, starch content, whose main function is to act as a reservoir for carbon and energy as it is insoluble, was also 1.8-fold higher in agonist-treated plants, suggesting that carbohydrate metabolism was enhanced by iSB09 (Fig. 5I).

### Transcriptional effect of ABA on genes involved in carbohydrate metabolism

**Fig. 6:**
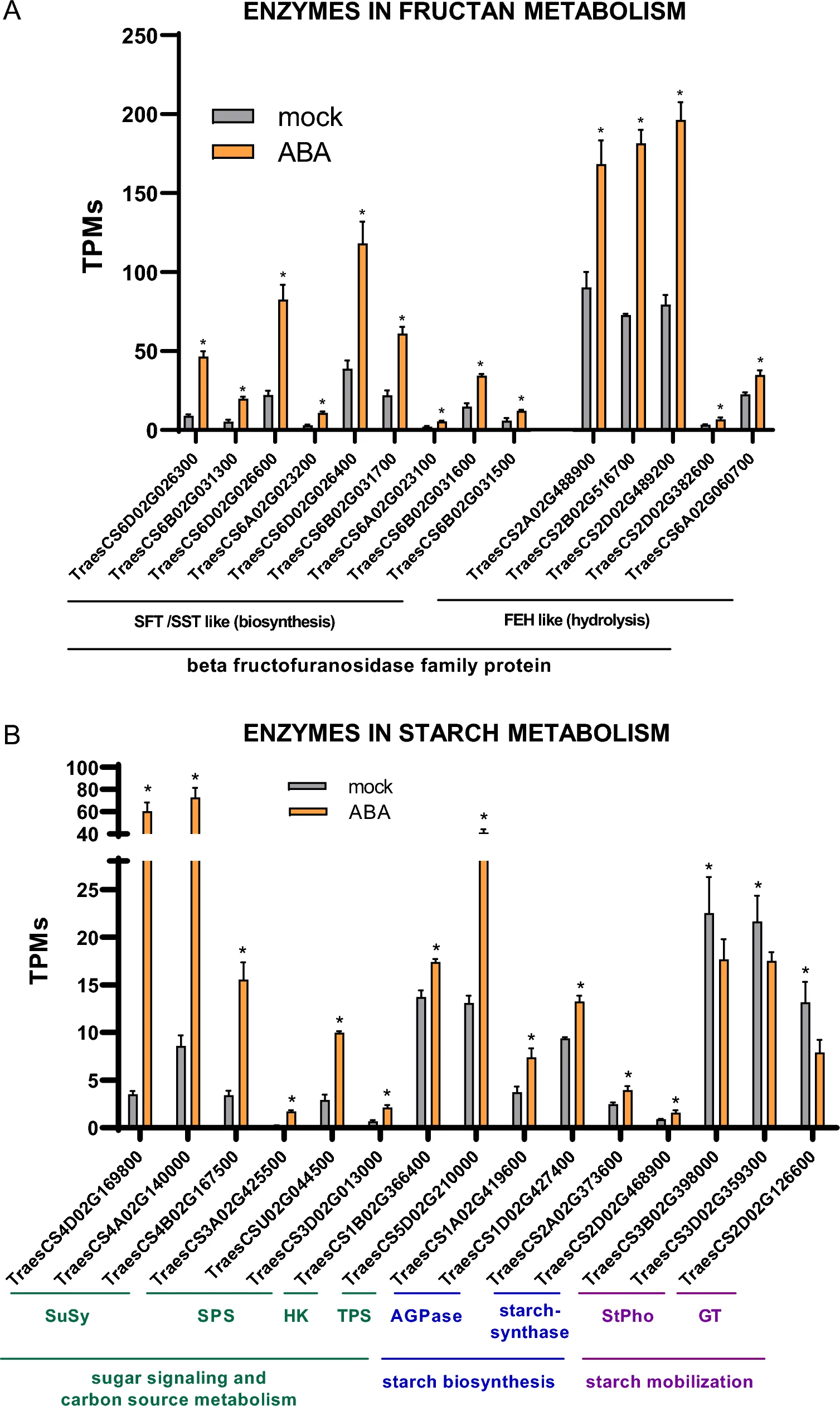
Activation of ABA signaling reprograms transcriptional response for carbohydrate metabolism. **A** RNA-seq analysis reveals that ABA treatment upregulates genes involved in fructan metabolism. Bars show the number of transcripts per million (TPMs) obtained from RNA-seq analysis of wheat plants mock-treated or treated with 20 μM ABA for 4 h. The indicated genes are involved either in fructan biosynthesis, such as sucrose:fructan fructosyltransferase (SFT) and sucrose:sucrose fructosylltransferase (SST), or fructan hydrolysis, such as fructan exohydrolases (FEH). Values are means of three biological replicates per treatment, and error bars represent the SD. Asterisks (*) indicate significant differences (*p* < 0.05). (B) RNA-seq analysis reveals that ABA treatment upregulates genes involved in starch metabolism. Bars show the TPMs in wheat plants for genes related to starch metabolism, such as sucrose synthases (Susy), sucrose-phosphate synthase (SPS), hexokinases (HK), trehalose-phosphate synthase (TPS), ADP-glucose pyrophosphorylase (AGPase), starch synthase, starch phosphorylase (StPho), and glucanotransferase (GT). Values are means of three biological replicates per treatment, and error bars represent the SD. Asterisks (*) indicate significant differences (*p* < 0.05)

We investigated the effect of ABA treatment on genes encoding enzymes involved in fructan metabolism using RNA-seq analysis. It has recently been reported that drought stress induces key genes involved in fructan metabolism, such as sucrose-sucrose 1-frutosyltransferase (1-SST), sucrose-fructan 6-fructosyltransferase (6-SFT), and fructan 1-exohydrolase (1-FEH) (Mostafaie et al. 2025; Vicente et al. 2024). This facilitates efficient short-term carbohydrate storage and carbon mobilization to supply sugars under stress conditions (Livingston et al. 2009; Mostafaie et al. 2025). We generated RNA-seq data from mock- or ABA-treated 7-day-old plants at 4 h post-treatment. We found that ABA treatment enhanced the expression of enzymes involved in fructan biosynthesis, namely 1-SST and 6-SFT (Fig. 6A). We also found upregulation of FEH-like enzymes, thus facilitating the mobilization of fructan reserves under abiotic stress conditions that increase ABA levels (Fig. 6A). Indeed, high-yielding wheat varieties under both optimal and water-stress conditions have been associated with greater fructan remobilization at the whole-plant level during grain filling (Vicente et al. 2024).

Concerning starch metabolism, we found that ABA induced upregulation of ADP-glucose pyrophosphorylase (AGPase), which catalyzes the first step in starch biosynthesis (Kim and Kim 2021) (Fig. 6B). Additionally, genes encoding enzymes involved in starch mobilization were downregulated (Fig. 6B). Other genes encoding enzymes involved in starch metabolism, such as sucrose phosphate synthase (SPS), sucrose synthase (SuSy), glucokinase, and starch synthase, were upregulated by ABA treatment (Fig. 6B). Interestingly, a gene encoding trehalose−6-phosphate synthase (T6P synthase) was upregulated by ABA treatment (Fig. 6B). T6P acts as a signal of sucrose status and a homeostatic feedback regulator, inhibiting an early step of starch degradation, and high T6P levels enhance starch biosynthesis in wheat (Fichtner 2025).

Finally, we analyzed genes encoding enzymes involved in the synthesis of the osmoprotective raffinose family of oligosaccharides (RFOs). Raffinose synthase (RS) gene family members in bread wheat have recently been systematically screened, and the overexpression of *TaRS15-3B* can improve the tolerance of transgenic bread wheat to drought and salt stresses (Guo et al. 2023). Interestingly, several *TaRS* genes were upregulated by ABA treatment, including *TaRS15-3B* (Supplementary Fig. 4). RS uses galactinol as a precursor, and there exists a correlation between the upregulation of galactinol synthase (GolS) and increased levels of galactinol and raffinose (Sengupta et al. 2015). Interestingly, in addition to RS, *TaGolS* genes were upregulated by ABA treatment (Supplementary Fig. 4).

## Discussion

### Wheat has 26 potential TaPYL ABA receptors and 12 LFS-like proteins

We have reexamined the extent of the PYL family of ABA receptors in wheat. Our results support the original identification of 26 members reported by Mega et al. (2019), but do not support recent publications that extend the family up to 38 members (Lei et al. 2021; Wang et al. 2025). Mega et al. (2019) provided biochemical evidence that TaPYL1-TaPYL9 are functional receptors in PP2C inhibition assays, specifically inhibiting TaPP2Cs in an ABA-dependent manner. Reliable identification of TaPYLs is crucial for several reasons, including harnessing their biotechnological potential to enhance drought tolerance (Mega et al. 2019; Mao et al. 2022). For example, the overexpression of TaPYL4 conferred a water-saving trait in wheat, reducing water consumption and leading to a concomitant increase in photosynthetic activity under water deficit (Mega et al. 2019). On the other hand, allelic variation in the promoter region of the TaPYL1 gene (1B subgenome) resulted in enhanced expression under drought stress and increased drought tolerance in selected wheat cultivars (Mao et al. 2022).

The 12 putative additional PYLs reported by the latter publications (Lei et al. 2021; Wang et al. 2025) are currently annotated as LFS-like proteins (Supplementary Figs. 1 and 2). Despite being annotated as LFS-like proteins, the most likely function of these proteins in wheat is not lachrymatory for the following reasons. Based on current knowledge, only onion LFS can produce the lachrymatory factor (*Z*)-propanethial *S*-oxide from a very specific precursor: (*E*)− 1-propenesulfenic acid (Silvaroli et al. 2017). This precursor is produced in onions by the action of alliinase on (*E*)-*S*-(1-propenyl)cysteine ​​*S*-oxide (Silvaroli et al. 2017). Such a metabolic pathway is absent in wheat, which lacks the profile of organic sulfur compounds from *Allium* species (e.g., onion, garlic). Therefore, it is doubtful that the specific substrate required for the formation of the lachrymatory factor is present in wheat.

### Structural and bioinformatic analyses reveal that LFS-like proteins belong to the polyketide cyclase protein superfamily, but cannot bind ABA

The 12 LFS-like proteins studied in this work belong to the Bet v/START/Polyketide cyclase protein superfamily, which is characterized by a structural fold that enables the binding of hydrophobic ligands (Radauer et al. 2008). These proteins exhibit the helix-grip fold, which consists of a central β-sheet flanked by two helices at the N- and C-termini (Iyer et al. 2001). All these proteins contain an internal hydrophobic cavity of elongated shape; however, Bet v/START/Polyketide cyclase proteins accommodate very different ligands, for example, plant steroids, alkaloids, ABA, the lipid monolinolenin, or the precursor of the lachrymatory factor (Markovic-Housley et al. 2003; Santiago et al. 2009; Silvaroli et al. 2017; Sterlin et al. 2019). Thus, structural studies of the LFS enzyme in onion revealed essential differences in the ligand-binding sites of LFS and PYL proteins (Silvaroli et al. 2017). For example, the total volume of the ligand-binding site in the apo-form of PYL proteins is over 500 Å^3^, whereas it is reduced to 216 Å^3^ in LFS (Silvaroli et al. 2017). This enables PYL proteins to accommodate relatively large ligands (e.g., ABA and ABA receptor agonists) compared with smaller LFS ligands, such as propenesulfenic acid. Our bioinformatic and structural analyses (Figs. 2 and 3) provide strong evidence that only the canonical TaPYLs, exemplified here by TaPYL1B, possess the architectural features required for ABA perception and PP2C inhibition. Thus, although both TaPYL1B and TraesCS3B02G160200 adopt the characteristic Bet v/START fold, only TaPYL1B conserves the gate and latch loops, the Lys residue needed to coordinate the ABA carboxylate, and the positioning of Pro109 and Arg137 necessary to assemble the Trp lock and bind to the phosphatase (Melcher et al. 2009) (Fig. 3). In contrast, TraesCS3B02G160200 displays steric clashes within the ligand-binding cavity, lacks the conserved Lys, and is predicted to be unable to form the ABA:PP2C ternary complex, which was confirmed by biochemical analyses (Fig. 4A and B). These structural constraints are incompatible with ABA binding and signaling, indicating that the 12 putative TaPYLs resemble Bet v/START family proteins, but they are not functional ABA receptors. This functional divergence suggests that wheat encodes a smaller set of true ABA receptors than previously proposed by Lei et al. (2021). Further biochemical validation and ligand screening are required to clarify the physiological roles of these Bet v/START-like wheat proteins.

### Chemical activation of ABA signaling by the ABA receptor agonist iSB09

Genetic approaches have previously highlighted the biotechnological potential of TaPYLs. In this work, we have explored a chemical approach to activate TaPYLs using the chemical ligand iSB09. Unlike genetic modification, chemical approaches offer on-demand control over ABA signaling. The development of ABA receptor agonists is necessary because exogenous ABA application is inefficient due to its UV sensitivity and short half-life (Gao et al. 2016; Han et al. 2017). In our hands, the antitranspirant effect of ABA, when applied to 40-d-old plants, was not evident at 72 h after application. In contrast, iSB09 showed a more persistent effect, and diminished gs and E even at 7 days after spraying. As a result, soil water consumption decreased, while biomass production remained unaffected, leading to increased water productivity (Fig. 5F and G). Interestingly, photosynthesis was not impaired by agonist treatment at 7 days after spraying, even with a lower Ci, resulting in higher WUE and iWUE in wheat (Fig. 5C). Recent studies have demonstrated that improving leaf water status via ABA signaling enhances leaf growth and water-use efficiency without impairing biomass production in *Arabidopsis* (Liu et al. 2025). Additionally, leaf growth is closely connected with plant hydraulics, and ABA regulates leaf and root water conductivity (Shahzad et al. 2024; Liu et al. 2025). Therefore, in addition to carbon availability, ABA-regulated leaf and root hydraulic conductivity, and leaf water status impact on biomass yield (Shahzad et al. 2024; Liu et al. 2025).

Agonist treatment had a positive effect on sugar metabolism, increasing both fructan and starch content. Fructans serve for short-term carbohydrate storage, and we found that ABA signaling upregulated the enzymes involved in fructan biosynthesis. Additionally, fructans play a critical role in abiotic stress tolerance, both by directly stabilizing membranes and by providing hexose sugars for plant growth (Livingston et al. 2009). Interestingly, ABA also stimulates fructan remobilization by upregulating FEH-like enzymes (Fig. 6A). Additionally, several *TaRS* and *TaGolS* genes were upregulated by ABA treatment, which can favor the synthesis of the osmoprotective RFOs (Sengupta et al. 2015; Salvi et al. 2020; Guo et al. 2023) (Supplementary Fig. 4). Therefore, ABA signaling might promote osmoprotection in wheat by stimulating fructan and raffinose synthesis. We validated by RT-qPCR analysis that iSB09 treatment also induces upregulation of genes involved in fructan and raffinose biosynthesis (Supplementary Fig. 5). This ABA-mediated pre-adaptation may provide the plant with a proactive biochemical shield, mitigating oxidative damage and helping to maintain cellular turgor prior to the onset of severe water deficit (Nishizawa et al. 2008; Livingston et al. 2009; Sengupta et al. 2015). Our study also demonstrated that activation of ABA signaling by iSB09 led to a 1.8-fold increase in starch accumulation. At the transcriptional level, ABA strongly induced the expression of AGPase, which catalyzes the first specific step in starch biosynthesis (Fig. 6B). Furthermore, genes involved in starch metabolism were upregulated following ABA treatment, whereas genes encoding enzymes involved in starch mobilization were downregulated. Specifically, ABA treatment upregulated T6P expression, a homeostatic feedback regulator that enhances starch biosynthesis in wheat (Fichtner et al. 2025). This suggests that ABA signaling promotes carbohydrate metabolism by accumulating starch and fructans as a temporary energy sink. Such a carbohydrate reserve could help mitigate the negative impacts of a subsequent water deficit, given the established roles of these compounds in osmoprotection and stress-induced remobilization (Kim and Kim 2021; Mostafaie et al. 2025). However, our experiments were conducted under non-stress, controlled conditions, and we did not measure drought tolerance, photosynthetic decline under water deficit, or yield-related traits; therefore, this connection remains a hypothesis for future testing under actual drought conditions. It is important to note that the physiological and biochemical experiments in this study were performed under well-watered, controlled conditions, without an actual drought stress episode. Therefore, our results demonstrate that iSB09 reduces water consumption and increases water productivity under these conditions, but they do not by themselves demonstrate improved drought tolerance or yield maintenance under water deficit.

## Conclusion

This study provides a refined census of the wheat TaPYL family, identifying 26 functional ABA receptors and clarifying that 12 previously proposed candidates lack essential structural features for ligand binding. By employing the synthetic agonist iSB09, we demonstrate a high-precision strategy for chemically activating ABA signaling that significantly outperforms exogenous ABA in terms of persistence. Treatment with iSB09 reduced water consumption by 25% without compromising biomass production, resulting in markedly higher water productivity. Beyond transpiration control, this study reveals a coordinated reprogramming of carbohydrate metabolism by the ABA pathway. Specifically, iSB09 treatment enhances the accumulation of starch and protective osmolytes, such as fructans. These findings establish iSB09 as a powerful chemical agonist for certain wheat ABA receptors, coupling antitranspirant effects with the metabolic reinforcement of carbohydrate reserves. This dual effect of reduced water consumption coupled with reinforced carbohydrate reserves represents a promising basis for improving water-use efficiency in wheat; whether it translates into improved performance under actual drought stress or into yield benefits remains to be tested directly.

## Supplementary Information

Below is the link to the electronic supplementary material.

Supplementary file1 (PDF 904 KB)

## Data Availability

All data supporting the findings of this study are available within the paper and within its supplementary data published online. Raw reads and differential expression gene tables used in this paper have been deposited in the Gene Expression Omnibus under accession number GSE317722.
