# Supplementary material for: TaPYL-mediated chemical activation of ABA signaling in wheat enhances water productivity and reinforces carbohydrate reserves
Source: Planta. 2026 Nov 10;264(6):190. doi: 10.1007/s00425-026-05216-3 (PMC13751870; doi:10.1007/s00425-026-05216-3)
Supplement: Supplementary file 1 [file 425_2026_5216_MOESM1_ESM.pdf]

A

Lei et al., 2021	Gene code (IWGSC v.1)
TaPYL26	TraesCS3D02G142500
TaPYL22	TraesCS3B02G160300
TaPYL18	TraesCS3A02G311900
TaPYL17	TraesCS3A02G285000
TaPYL28	TraesCS3D02G284800
TaPYL23	TraesCS3B02G318900
TaPYL10	TraesCS1D02G127600
TaPYL1	TraesCS1A02G125900
TaPYL5	TraesCS1B02G145000
TaPYL19	TraesCS3A02G312000
TaPYL21	TraesCS3B02G160200
TaPYL25	TraesCS3D02G142400

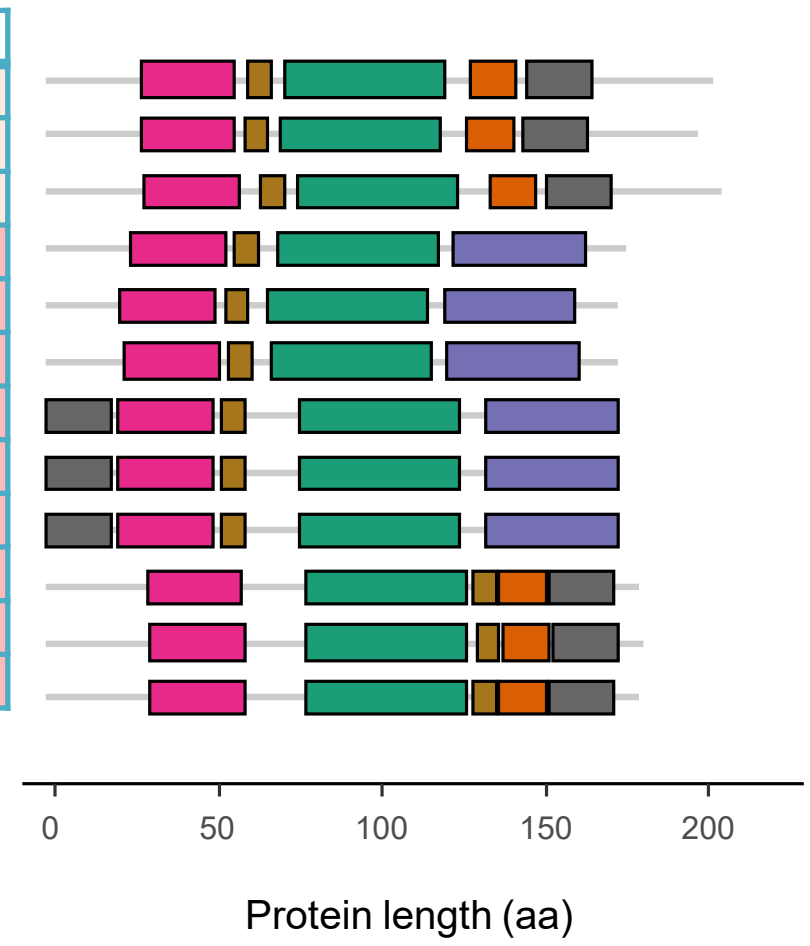

B

Mega et al., 2019	Gene code (IWGSC v.1)
TaPYL9-D	TraesCS7D02G328000
TaPYL9-A	TraesCS7A02G350800
TaPYL9-B	TraesCS7B02G232100
TaPYL8-A	TraesCS1A02G126800
TaPYL8-D	TraesCS1D02G126900
TaPYL8-B	TraesCS1B02G145800
TaPYL7-A	TraesCS4A02G114400
TaPYL7-D	TraesCS4D02G191200
TaPYL7-B	TraesCS4B02G189800
TaPYL4-D	TraesCS2D02G087500
TaPYL4-B	TraesCS2B02G105300
TaPYL4-A	TraesCS2A02G089400
TaPYL6-D	TraesCS1D02G293600
TaPYL6-B	TraesCS1B02G306800
TaPYL6-A	TraesCS1A02G297600
TaPYL5-A	TraesCS3A02G348400
TaPYL5-B	TraesCS3B02G380300
TaPYL5-D	TraesCS3D02G342000
TaPYL1-D	TraesCS1D02G195300
TaPYL1-A	TraesCS1A02G191700
TaPYL1-B	TraesCS1B02G206600
TaPYL2-A	TraesCS3A02G154400
TaPYL2-D	TraesCS3D02G161500
TaPYL3-D	TraesCS7D02G364600
TaPYL3-B	TraesCS7B02G269600
TaPYL3-A	TraesCS7A02G358200

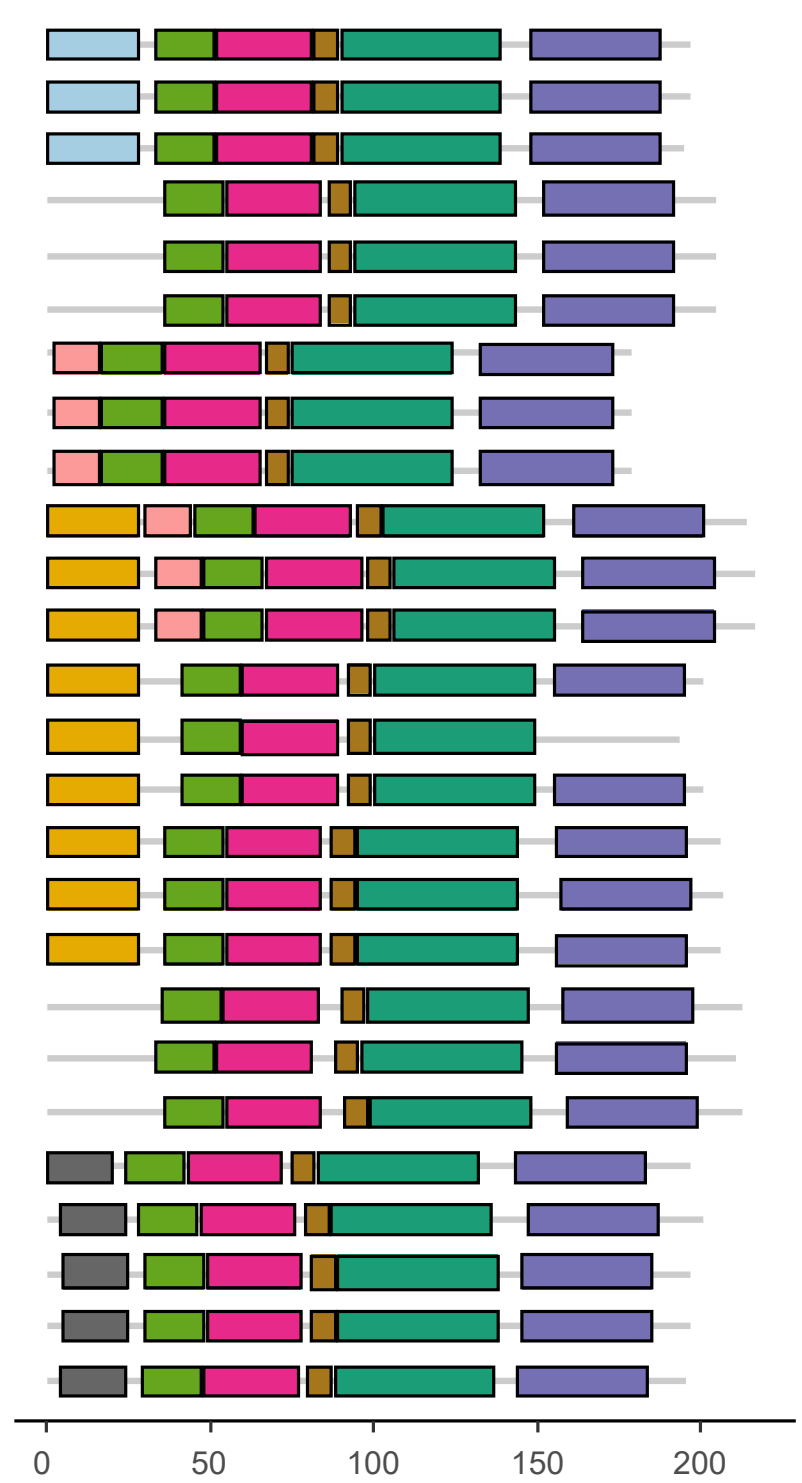

C

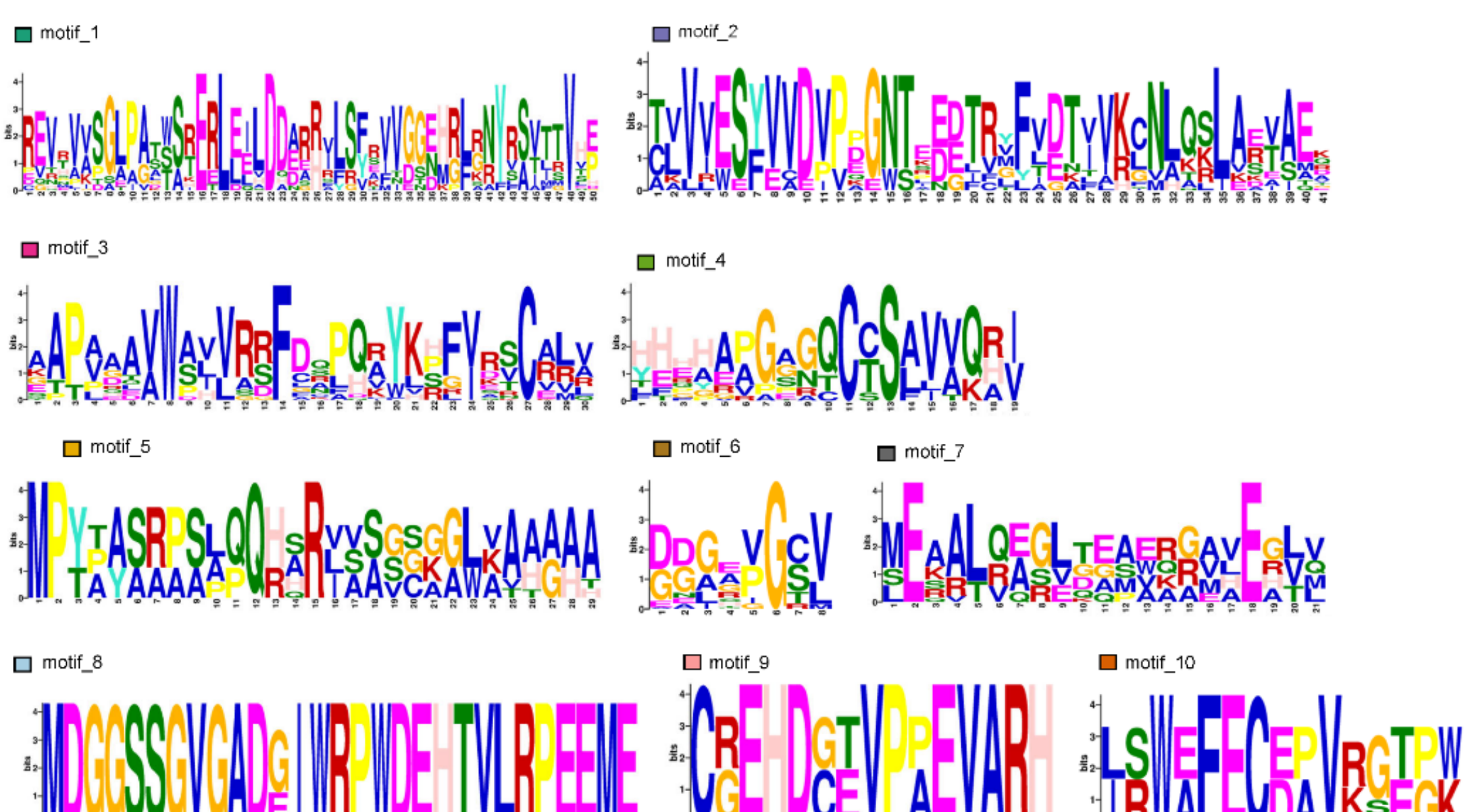

Supplementary Figure 1. Traes nomenclature of the 38 bread wheat Polyketide_cyc2 PFAM-containing proteins. The MEME suite analysis reveals divergence of key residues for ABA binding between cluster 1 canonical TaPYLs and cluster 2/3 proteins

(A) Cluster 2 and 3 genes. (B) Cluster 1 canonical TaPYLs. (C) To explore sequence motifs in the 38 bread wheat Polyketide_cyc2 PFAM-containing proteins, the MEME suite was used with the -mod zoops mode, and ten conserved motifs were identified. Motifs are numbered from 1 to 10 and are represented by different colors. Sequence logos corresponding to each identified motif are shown. Motif_3 includes the conserved Gate and Latch motifs in canonical TaPYL proteins, each spanning 3 amino acids. Gate and latch motifs are conserved in canonical TaPYL proteins (except in TaPYL9 for the latch) but show variation in BetV/START-type proteins, reflecting differences in key functional sites between the two groups.

Supplemental Figure 2

BLASTp of TraesCS3B02G160300

Protein Name	Organism (Full Name)	Accession	Score	% Identity	E-value
lachrymatory-factor synthase-like	Triticum dicoccoides	XP_037412087.1	402	100.00	2,00E-141
lachrymatory-factor synthase	Aegilops tauschii	XP_020171406.1	353	92.65	9,00E-122
lachrymatory-factor synthase-like	Hordeum vulgare	XP_044972236.1	333	89.32	8,00E-114
lachrymatory-factor synthase-like	Triticum dicoccoides	XP_037404734.1	310	86.96	6,00E-105
lachrymatory-factor synthase-like	Triticum urartu	XP_048562340.1	308	86.47	3,00E-104
lachrymatory-factor synthase-like	Lolium rigidum	XP_047052843.1	280	79.47	3,00E-93
lachrymatory-factor synthase-like	Phragmites australis	XP_062208374.1	280	76.22	6,00E-93
lachrymatory-factor synthase-like	Lolium rigidum	XP_047053331.1	275	78.01	4,00E-91
lachrymatory-factor synthase-like	Lolium perenne	XP_051223728.1	275	80.00	6,00E-91
lachrymatory-factor synthase-like	Lolium rigidum	XP_047057554.1	268	85.53	2,00E-88
lachrymatory-factor synthase	Setaria italica	XP_004968540.1	268	75.66	4,00E-88
lachrymatory-factor synthase	Setaria viridis	XP_034593529.1	268	75.66	4,00E-88
lachrymatory-factor synthase-like	Phragmites australis	XP_062182726.1	266	74.33	2,00E-87
lachrymatory-factor synthase-like	Lolium perenne	XP_051223727.1	264	76.44	5,00E-86
lachrymatory-factor synthase	Brachypodium distachyon	XP_014753811.1	262	68.66	1,00E-85
lachrymatory-factor synthase	Sorghum bicolor	XP_002457176.1	257	73.89	6,00E-84
lachrymatory-factor synthase-like	Lolium rigidum	XP_047058293.1	255	75.26	4,00E-83
lachrymatory-factor synthase-like	Oryza glaberrima	XP_052151276.1	252	73.74	1,00E-81
lachrymatory-factor synthase-like	Oryza brachyantha	XP_040385595.1	249	72.34	8,00E-81
lachrymatory-factor synthase	Oryza sativa	XP_015626787.1	248	71.72	4,00E-80
lachrymatory-factor synthase-like	Miscanthus floridulus	XP_066305558.1	243	70.41	4,00E-78
lachrymatory-factor synthase-like	Panicum hallii	XP_025814007.1	237	74.19	4,00E-76
Lachrymatory-factor synthase-like	Zea mays	NP_001141298.1	236	71.81	1,00E-75
lachrymatory-factor synthase	Brachypodium distachyon	XP_003566442.2	224	67.20	6,00E-71
lachrymatory-factor synthase-like	Lolium rigidum	XP_047043222.1	223	65.95	2,00E-70
lachrymatory-factor synthase-like	Panicum virgatum	XP_039813391.1	219	66.67	4,00E-69
lachrymatory-factor synthase-like	Lolium rigidum	XP_047043219.1	218	65.41	1,00E-68
lachrymatory-factor synthase	Lolium perenne	XP_051181793.1	217	64.32	2,00E-68
lachrymatory-factor synthase-like	Panicum virgatum	XP_039846099.1	214	68.95	4,00E-67
lachrymatory-factor synthase-like	Oryza glaberrima	XP_052156665.1	190	60.61	1,00E-57
lachrymatory-factor synthase	Oryza sativa Japonica	XP_015638934.1	188	62.18	1,00E-56
lachrymatory-factor synthase-like	Oryza brachyantha	XP_040380483.1	183	63.79	6,00E-55
lachrymatory-factor synthase-like	Musa acuminata	XP_009405224.1	144	46.67	4,00E-40
lachrymatory-factor synthase-like	Musa acuminata	XP_064966948.1	142	46.00	3,00E-39
lachrymatory-factor synthase-like	Phoenix dactylifera	XP_008795127.3	140	41.62	1,00E-38

BLASTp of TraesCS3B02G318900

Protein Name	Organism	Accession	Score	% identity	E-value
lachrymatory-factor synthase-like	Triticum dicoccoides	XP_037417903.1	351	98.86	5,00E-122
lachrymatory-factor synthase-like	Triticum dicoccoides	XP_037406585.1	329	93.26	2,00E-113
lachrymatory-factor synthase-like	Triticum urartu	XP_048567714.1	329	92.70	3,00E-113
lachrymatory-factor synthase	Aegilops tauschii	XP_020161881.1	327	93.18	1,00E-112
lachrymatory-factor synthase-like	Hordeum vulgare	XP_044972699.1	321	90.86	4,00E-110
lachrymatory-factor synthase-like	Lolium rigidum	XP_047056862.1	286	88.37	4,00E-96
lachrymatory-factor synthase	Lolium perenne	XP_051225774.1	275	87.13	8,00E-92
lachrymatory-factor synthase	Brachypodium distachyon	XP_003567065.1	274	81.14	1,00E-91
lachrymatory-factor synthase-like	Oryza brachyantha	XP_006646278.1	264	74.57	4,00E-87
lachrymatory-factor synthase-like	Phragmites australis	XP_062220075.1	261	82.35	2,00E-86
Lachrymatory-factor synthase	Zea mays	NP_001152295.1	246	72.41	2,00E-80
lachrymatory-factor synthase-like	Miscanthus floridulus	XP_066306762.1	245	73.84	5,00E-80
lachrymatory-factor synthase-like	Miscanthus floridulus	XP_066309566.1	242	70.69	1,00E-78
lachrymatory-factor synthase-like	Oryza glaberrima	XP_052152906.1	241	73.58	1,00E-77
lachrymatory-factor synthase	Oryza sativa	XP_015642869.1	239	72.96	4,00E-77
lachrymatory-factor synthase-like	Panicum hallii	XP_025813982.1	225	76.92	3,00E-72
lachrymatory-factor synthase-like	Typha angustifolia	XP_072967832.1	223	66.03	2,00E-71
lachrymatory-factor synthase	Setaria viridis	XP_034595733.1	220	68.57	3,00E-70
lachrymatory-factor synthase	Setaria italica	XP_004969803.1	220	68.00	4,00E-70
lachrymatory-factor synthase-like	Typha latifolia	XP_072995528.1	219	65.38	6,00E-70
lachrymatory-factor synthase	Ananas comosus	XP_020099215.1	207	64.90	4,00E-65
lachrymatory-factor synthase	Phoenix dactylifera	XP_008804606.1	204	60.87	4,00E-64
lachrymatory-factor synthase-like	Musa acuminata	XP_065023076.1	203	62.18	2,00E-63
lachrymatory-factor synthase-like	Panicum virgatum	XP_039813045.1	200	66.87	3,00E-62
lachrymatory-factor synthase	Musa acuminata	XP_064938189.1	197	60.90	3,00E-61
lachrymatory-factor synthase	Dioscorea cayenensis	XP_039116391.1	180	53.21	1,00E-54
lachrymatory-factor synthase	Magnolia sinica	XP_058115115.1	150	44.94	9,00E-43
lachrymatory-factor synthase-like	Cornus florida	XP_059643153.1	148	42.59	5,00E-42
lachrymatory-factor synthase-like	Tasmannia lanceolata	XP_077254020.1	145	43.79	8,00E-41
lachrymatory-factor synthase-like	Carya illinoinensis	XP_042974220.1	142	42.42	2,00E-39
lachrymatory-factor synthase-like	Juglans regia	XP_018832530.2	141	43.79	3,00E-39
lachrymatory-factor synthase-like	Raphanus sativus	XP_018482112.1	140	45.40	1,00E-38
lachrymatory-factor synthase	Daucus carota	XP_017221100.1	140	38.04	1,00E-38
lachrymatory-factor synthase-like	Juglans regia	XP_035538534.1	140	43.14	1,00E-38

BLASTp of TraesCS1B02G145000

Protein Name	Organism	Accession	Score	% identity	E -value
lachrymatory-factor synthase	Aegilops tauschii	XP_020176049.1	348	97.73	8,00E-121
lachrymatory-factor synthase	Lolium perenne	XP_051181805.1	259	78.74	9,00E-86
lachrymatory-factor synthase-like	Lolium rigidum	XP_047043223.1	254	78.03	1,00E-83
lachrymatory-factor synthase-like	Lolium rigidum	XP_047043220.1	248	75.72	2,00E-81
lachrymatory-factor synthase-like	Oryza brachyantha	XP_040380514.1	228	66.48	3,00E-72
lachrymatory-factor synthase-like	Phragmites australis	XP_062230995.1	218	65.50	2,00E-69
lachrymatory-factor synthase-like	Panicum virgatum	XP_039836201.1	211	64.63	4,00E-66
lachrymatory-factor synthase	Setaria viridis	XP_034584535.1	209	64.63	2,00E-65
lachrymatory-factor synthase	Setaria italica	XP_004960597.1	209	64.63	2,00E-65
lachrymatory-factor synthase-like	Miscanthus floridulus	XP_066366807.1	207	62.87	6,00E-65
lachrymatory-factor synthase-like	Phragmites australis	XP_062180509.1	203	69.33	2,00E-63
lachrymatory-factor synthase-like	Panicum hallii	XP_025807785.1	205	66.23	3,00E-63
lachrymatory factor synthase	Zea mays	NP_001151021.2	196	64.90	2,00E-60
lachrymatory-factor synthase-like	Panicum virgatum	XP_039803050.1	193	62.50	2,00E-59
lachrymatory-factor synthase-like	Panicum virgatum	XP_039803048.1	183	61.64	2,00E-55
lachrymatory-factor synthase-like	Oryza glaberrima	XP_052140618.1	181	51.72	9,00E-55
lachrymatory-factor synthase-like	Typha angustifolia	XP_072979596.1	164	50.00	4,00E-48
lachrymatory-factor synthase-like	Typha latifolia	XP_073001977.1	161	50.30	5,00E-47
lachrymatory-factor synthase-like	Phoenix dactylifera	XP_008794116.1	158	47.02	8,00E-46
lachrymatory-factor synthase-like	Oryza brachyantha	XP_040376025.1	158	53.80	2,00E-45
lachrymatory-factor synthase	Musa acuminata	XP_009404534.1	144	42.26	2,00E-40
lachrymatory-factor synthase-like	Oryza brachyantha	XP_006646278.1	144	46.25	6,00E-40
lachrymatory-factor synthase-like	Musa acuminata	XP_065045873.1	144	42.35	1,00E-39
lachrymatory-factor synthase-like	Phoenix dactylifera	XP_008795127.3	140	43.11	1,00E-38
lachrymatory-factor synthase	Oryza sativa	XP_015642869.1	138	41.52	2,00E-37
lachrymatory-factor synthase	Elaeis guineensis	XP_010915407.2	136	41.92	5,00E-37
lachrymatory-factor synthase-like	Oryza glaberrima	XP_052152906.1	137	40.94	5,00E-37
lachrymatory-factor synthase-like	Musa acuminata	XP_064966948.1	134	42.42	2,00E-36
lachrymatory-factor synthase-like	Musa acuminata	XP_009405224.1	134	42.42	3,00E-36
lachrymatory-factor synthase	Magnolia sinica	XP_058115115.1	132	38.55	1,00E-35
lachrymatory-factor synthase-like	Curcuma longa	XP_074565875.1	131	44.31	4,00E-35
lachrymatory-factor synthase-like	Triticum dicoccoides	XP_037417903.1	131	43.10	4,00E-35
lachrymatory-factor synthase	Daucus carota	XP_017221100.1	130	37.50	5,00E-35
lachrymatory-factor synthase-like	Zingiber officinale	XP_042432821.1	131	42.35	6,00E-35

BLASTp of TraesCS3B02G160200

Description	Description2	Accession	Score	% identity	E value
lachrymatory-factor synthase-like	Oryza brachyantha	XP_040376025.1	229	73.33	1,00E-73
lachrymatory-factor synthase-like	Oryza glaberrima	XP_052140618.1	164	53.09	7,00E-48
lachrymatory-factor synthase-like	Phragmites australis	XP_062230995.1	163	54.82	8,00E-48
lachrymatory-factor synthase-like	Lolium rigidum	XP_047043223.1	158	53.98	2,00E-45
lachrymatory-factor synthase-like	Lolium rigidum	XP_047043220.1	154	53.85	4,00E-44
lachrymatory-factor synthase	Lolium perenne	XP_051181805.1	153	52.25	9,00E-44
lachrymatory-factor synthase	Aegilops tauschii	XP_020176049.1	153	52.57	1,00E-43
lachrymatory-factor synthase-like	Miscanthus floridulus	XP_066366807.1	152	50.29	3,00E-43
lachrymatory-factor synthase-like	Panicum virgatum	XP_039836201.1	147	52.05	4,00E-41
lachrymatory-factor synthase	Setaria viridis	XP_034584535.1	145	54.09	3,00E-40
lachrymatory-factor synthase	Setaria italica	XP_004960597.1	145	54.09	4,00E-40
lachrymatory factor synthase	Zea mays	NP_001151021.2	142	55.26	3,00E-39
lachrymatory-factor synthase-like	Oryza brachyantha	XP_040380514.1	144	49.47	4,00E-39
lachrymatory-factor synthase-like	Phragmites australis	XP_062180509.1	141	54.36	4,00E-39
lachrymatory-factor synthase-like	Panicum hallii	XP_025807785.1	142	53.46	2,00E-38
lachrymatory-factor synthase-like	Panicum virgatum	XP_039803050.1	138	49.43	1,00E-37
lachrymatory-factor synthase-like	Typha latifolia	XP_073001977.1	132	43.48	9,00E-36
lachrymatory-factor synthase-like	Typha angustifolia	XP_072979596.1	132	42.86	3,00E-35
lachrymatory-factor synthase-like	Panicum virgatum	XP_039803048.1	131	50.30	7,00E-35
lachrymatory-factor synthase-like	Phoenix dactylifera	XP_008795127.3	127	40.74	9,00E-34
lachrymatory-factor synthase-like	Oryza glaberrima	XP_052156832.1	128	45.45	2,00E-33
lachrymatory-factor synthase-like	Phoenix dactylifera	XP_008794116.1	126	41.61	3,00E-33
lachrymatory-factor synthase	Musa acuminata	XP_009404534.1	125	42.86	6,00E-33
lachrymatory-factor synthase-like	Musa acuminata	XP_009405224.1	125	41.92	8,00E-33
lachrymatory-factor synthase-like	Musa acuminata	XP_064966948.1	125	41.67	9,00E-33
lachrymatory-factor synthase-like	Musa acuminata	XP_065045873.1	125	42.86	3,00E-32
lachrymatory-factor synthase	Elaeis guineensis	XP_010915407.2	123	40.12	6,00E-32
lachrymatory-factor synthase-like	Curcuma longa	XP_074565875.1	118	42.59	8,00E-30
lachrymatory-factor synthase-like	Zingiber officinale	XP_042432821.1	117	44.10	2,00E-29
lachrymatory-factor synthase-like	Syzygium oleosum	XP_030452761.1	111	35.67	2,00E-27
lachrymatory-factor synthase-like	Panicum hallii	XP_025813982.1	110	41.72	7,00E-27
lachrymatory-factor synthase-like	Salvia splendens	XP_042037665.1	109	40.88	8,00E-27
lachrymatory-factor synthase	Lolium perenne	XP_051181793.1	109	40.83	2,00E-26
lachrymatory-factor synthase-like	Ananas comosus	XP_020097659.1	107	45.39	1,00E-25
lachrymatory-factor synthase	Morus notabilis	XP_010102414.1	107	35.54	2,00E-25

Supplementary Figure 2. BLASTp of TraesCS3B02G160300, TraesCS3B02G318900, TraesCS1B02G145000, and TraesCS3B02G160200 reveals similarity to proteins annotated as LFS or LFS-like. BLASTp search of the indicated protein sequence against the RefSeq Select proteins database (https://blast.ncbi.nlm.nih.gov/Blast.cgi), excluding *Triticum aestivum.* Only proteins with functional annotations are shown; hits labeled as "uncharacterized" or "predicted" were excluded from the final dataset for clarity.

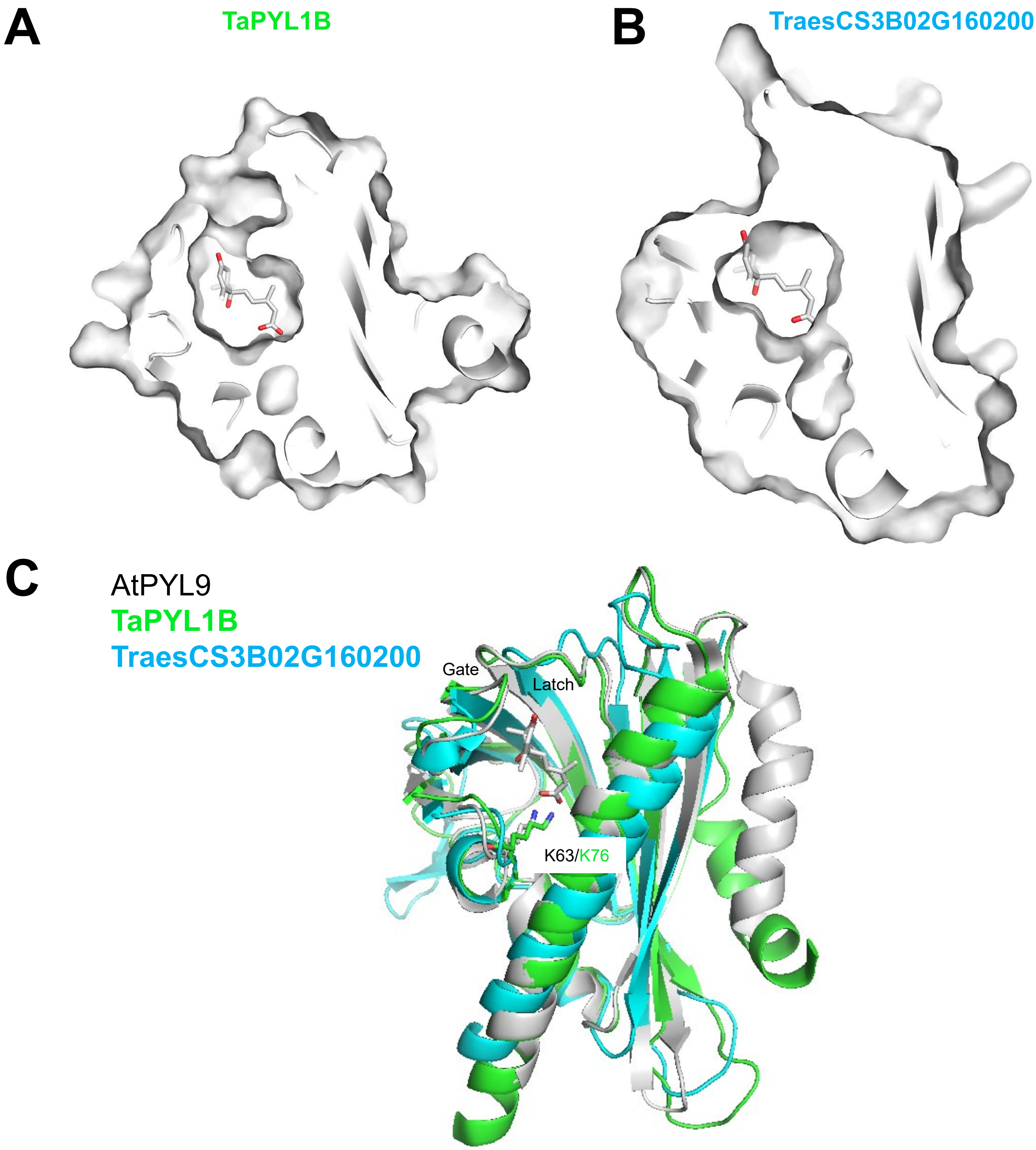

Supplementary Figure 3. The polyketide_cyc2 TraesCS3B02G160200 protein cannot accommodate ABA into the ligand-binding cavity. Sections of a surface representation of the ligand binding cavity in the predicted structures of TaPYL1B (A) and TraesCS3B02G160200 (B). While ABA fits into the cavity of TaPYL1B, it protrudes into the ligand-binding cavity of TraesCS3B02G160200, thereby hindering ABA binding. (C) Ribbon representation of the predicted structures of TaPYL1B and TraesCS3B02G160200 superimposed on the crystal structure of the AtPYL9-ABA complex. The ABA molecule can only form the Trp lock in AtPYL9 and TaPYL1B.

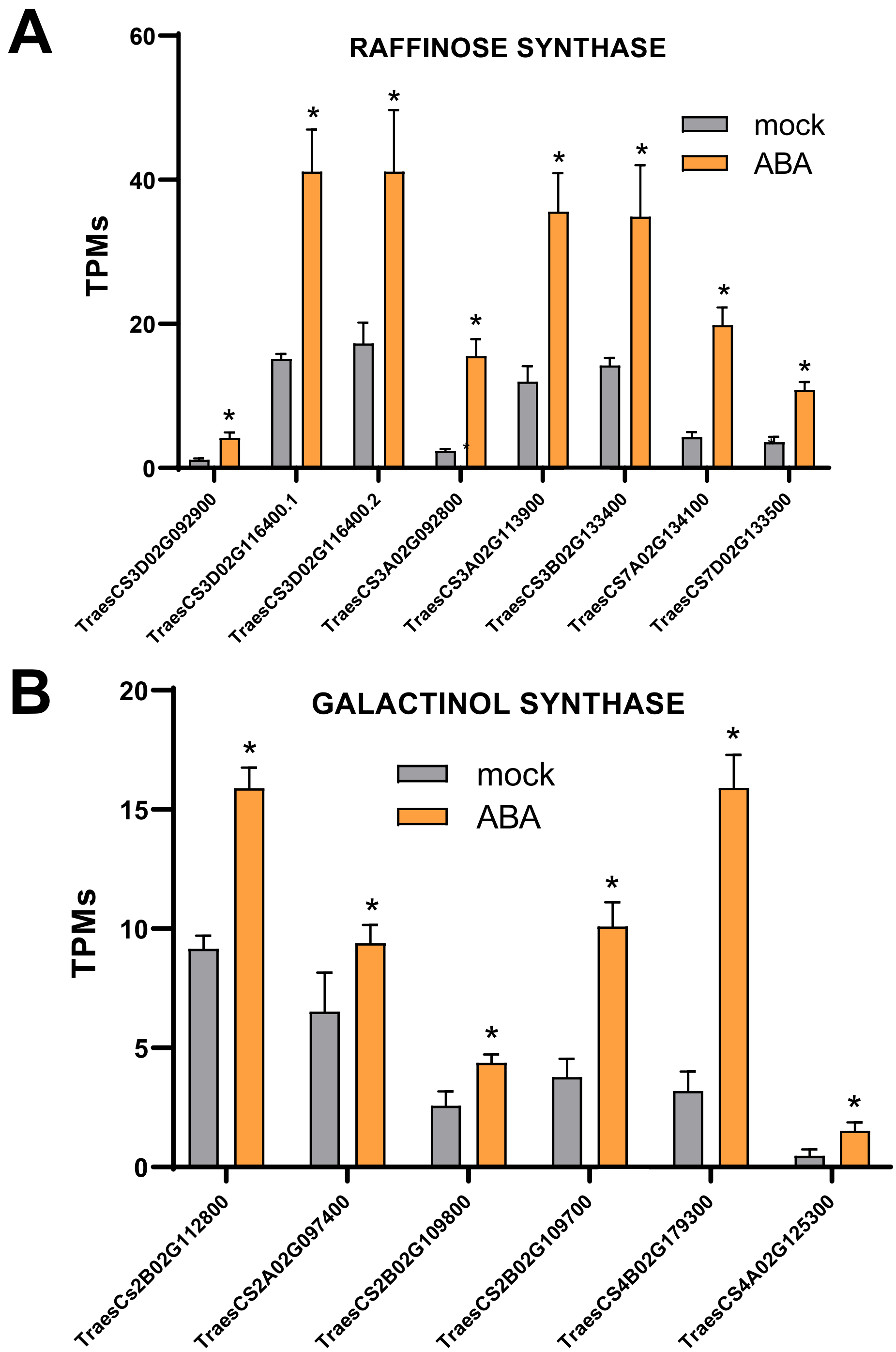

Supplementary Figure 4. Genes encoding galactinol and raffinose synthase are upregulated by ABA treatment.

(A) TPMs obtained via RNA-seq analysis. Different genes encoding raffinose synthase (EC 2.4.1.82) are upregulated in response to ABA treatment compared with mock-treated samples. The Traes genes indicated were previously identified by Guo et al., (2023) as raffinose synthases, and named (in order of appearance in the graph) as *TaRS18-3D*, *TaRS17-3D* (two transcripts), *TaRS13-3A*, *TaRS14-3A*, *TaRS15-3B*, *TaRS28-7A*, *TaRS33-7*. Bars represent the mean TPMs ± SD across three biological replicates per treatment. Asterisks (*) indicate significant differences ($p < 0.05$). (B) Different genes encoding galactinol synthase (EC: 2.4.1.123) are upregulated by ABA treatment. The Traes genes indicated were previously identified by Wang et al. (2016) as galactinol synthases, and named (in order of appearance in the graph)as *TaGolS1*, *TaGolS3*, *TaGolS5*, *TaGolS6*, *TaGolS7,* and *TaGolS8*.

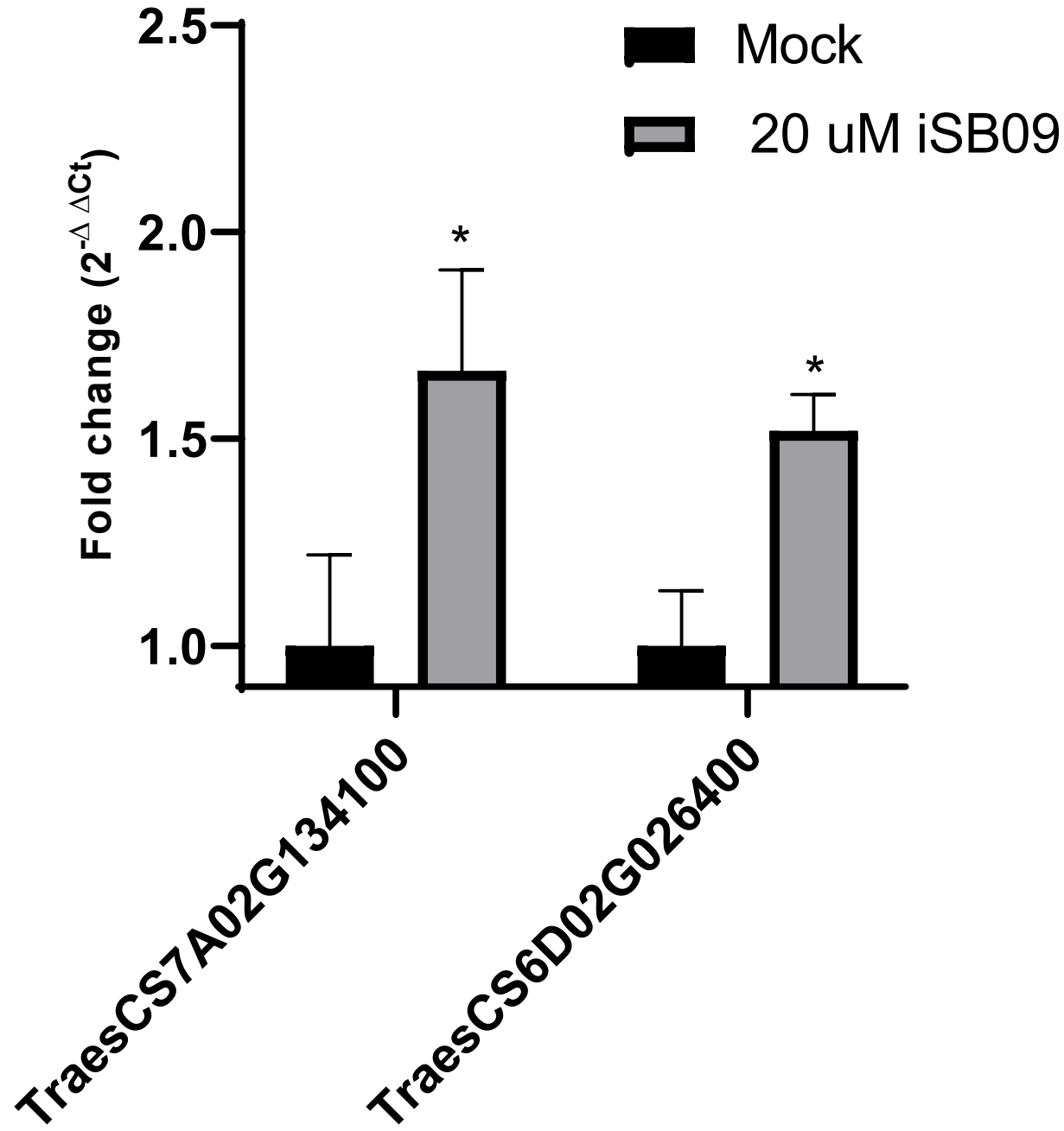

Supplementary Figure 5. Genes encoding enzymes involved in fructans or raffinose biosynthesis are upregulated by 20 μM iSB09 treatment.

Real-time qPCR analysis of *TraesCS6D02G026400* (involved in fructans biosynthesis) and *TraesCS7A02G134100* (involved in raffinose biosynthesis). Expression was normalized using the values obtained with the cyclophilin *TraesCS7A02G410100.* Relative quantification of gene expression was carried out using the comparative Ct method. Bars represent the mean ± SD across three biological replicates per treatment. Asterisks (*) indicate significant differences ($p < 0.05$).
